# Faster and Safer “In situ” Synthesis of Germanane and Silicane

**DOI:** 10.1002/smtd.202400964

**Published:** 2024-10-12

**Authors:** Yiannis Georgantas, Theodosis Giousis, Francis P. Moissinac, Gareth R. Tainton, Sarah J. Haigh, Mark A. Bissett

**Affiliations:** ^1^ Department of Materials Henry Royce Institute National Graphene Institute University of Manchester Ox‐ford Road Manchester M139PL UK; ^2^ Department of Materials Science & Engineering University of Ioannina Ioannina 45110 Greece; ^3^ Zernike Institute for Advanced Materials University of Groningen Nijenborgh 4 Groningen 9747 AG The Netherlands

**Keywords:** 2D materials, germanane, “in situ” HF, silicane, synthesis, Xanes

## Abstract

Germanane (GeH) and silicane (SiH), members of the Xanes family, have garnered significant attention as 2D materials due to their diverse properties, which hold promise for applications in electronics, optoelectronics, energy storage, and sensing. Typically, highly concentrated hydrochloric acid (HCl) or hydrofluoric acid (HF) is employed in the synthesis of these Xanes, but both routes are problematic due to slow kinetics and safety concerns, respectively. Here for the first time, a faster and safer method is demonstrated for Xanes synthesis that harnesses the generation of HF “in situ” using a solution of HCl and lithium fluoride (LiF) salt, overcoming the key challenges of the conventional methods. A variety of characterization techniques to establish a baseline is utilized for both Xanes and to provide a holistic knowledge regarding this method, the possible consequences of this approach, and the possibility of applying it to other layered Zintl phases. The novel synthesis protocol results in high‐quality GeH and SiH with bandgaps (*E*_g_) of 1.75 and 2.47 eV respectively, highlighting their potential suitability for integration into semiconductor applications.

## Graphical abstract

The work presents a safer, faster “in situ” method for synthesizing germanane and silicane using hydrochloric acid and lithium fluoride. It addresses safety and kinetic limitations of conventional methods. The materials show high crystallinity and purity, with germanane having a band gap of 1.75 eV and silicane 2.47 eV, making them suitable for electronics, optoelectronics, and energy storage applications.

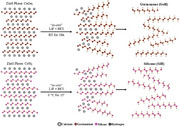

## 1 Introduction

Over the last decade, 2D materials (2DMs) have garnered significant attention and have become a focal point within the scientific community. The Xenes and Xanes families of 2D materials have attracted significant attention within the materials science and engineering community as a result of their unique electronic properties, structural diversity, and potential applications in various fields such as electronics,^[^ ^1^ ^]^ optoelectronics,^[^ ^2^ ^]^ energy storage,^[^ ^3^ ^]^ and catalysis.^[^ ^4^ ^]^

The Xenes, are structurally analogous to graphene, but carbon is replaced by other elements. Where those elements are from the 14^th^ group in the periodic table: e.g. silicon (Si), germanium (Ge), tin (Sn), and lead (Pb), this gives rise to silicene,^[^ ^5^ ^]^ germanene,^[^ ^6^ ^]^ stanene,^[^ ^7^ ^]^ and plumbene,^[^ ^8^ ^]^ respectively. Xenes have also been demonstrated with group 13 elements (boron and gallium), group 15 elements (phosphorus, arsenic, antimony, and bismuth), and group 16 elements (selenium and tellurium).^[^ ^9^ ^]^ The Xanes are hydrogen‐ or ligand‐functionalized derivatives of Xenes. Silicene (Si Xene), was first fabricated via molecular beam epitaxy (MBE) on a silver (111) substrate.^[^ ^5^, ^10^ ^]^ Subsequently, tellurene (Te Xene)^[^ ^11^ ^]^ and germanene (Ge Xene)^[^ ^6^ ^]^ were synthesized in 2014, marking the inception of the Xenes family. This expanded further with the introduction of stanene (Sn Xene)^[^ ^7^ ^]^ and borophene (B Xene)^[^ ^12^ ^]^ a year later.

While most Xenes and Xanes have only been synthesized using MBE, hydrogenated germanene (Germanane – GeH) and hydrogenated silicene (Silicane – SiH) Xanes can be synthesized via wet chemistry methods. To circumvent the bottom‐up challenges encountered in electron beam melting (EBM) fabrication processes for Xenes and Xanes, a top‐down approach was adopted first in 2001, involving topochemical deintercalation of β‐CaGe_2_ with hydrochloric acid (HCl) at low temperatures to yield layered GeH.^[^ ^13^ ^]^ Wet chemistry synthesis of silicane is performed using CaSi_2_ in concentrated HCl at low temperatures. This method was initially reported to produce Si_6_H_3_(OH)_3_ sheets^[^ ^14^ ^]^ known as siloxene (Kautsky‐type) with the Si_6_H_6_ structure reported in 1993.^[^ ^15^ ^]^ Despite these structural insights, the current literature lacks definitive evidence to comprehensively characterize the structure and coordination chemistry of these silicon nanosheets.

A common feature shared by both germanane and silicane is their reliance on Zintl phase precursors, specifically CaGe_2_ and CaSi_2_, respectively. Within the vast domain of Zintl phases, several exhibit a layered structure, with distinct layers formed by the anionic component separated from the interatomic cations. Moreover, a variety of methods exist for manipulating the structural dimensionality of Zintl phases deficient of such atomic layering to produce these distinct layers.^[^ ^16^ ^]^ Consequently, Zintl phases represent a promising platform for the top‐down synthesis of 2D materials.

The initial synthesis of GeH from β‐CaGe_2_ with HCl required temperatures ranging from −40 to −20 °C and reaction durations of 2–10 days.^[^ ^4^, ^17^ ^]^ To address these kinetic challenges, HCl was substituted for hydrofluoric acid (HF), significantly reducing the reaction time to a few minutes at room temperature.^[^ ^18^ ^]^ Analogously, MXenes are synthesized using similar principles, where the precursor is a layered transition metal carbide and/or nitride, separated by interatomic layers, primarily aluminum (Al). HF is employed to selectively etch away these interatomic layers, thereby isolating the 2D structure of MXenes.^[^ ^19^ ^]^ However, the direct use of HF entails various risks, including potential exposure leading to severe chemical burns, tissue damage, and, in extreme cases, fatality.

Herein, we demonstrate a safer approach involving the utilization of HCl and fluoride salt within the vessel to generate HF “in situ” for the etching process.^[^ ^20^ ^]^ The method demonstrated relatively rapid reaction kinetics, taking only several minutes to reach good product yield. Transmission electron microscopy techniques coupled with energy dispersive spectroscopy, X‐ray photoelectron spectroscopy, FT‐IR, and Raman spectroscopy have provided conclusive evidence of highly crystalline and low‐defect. GeH and SiH.

## 2 Results and Discussion

### 2.1 Precursors

**Figure 1:**
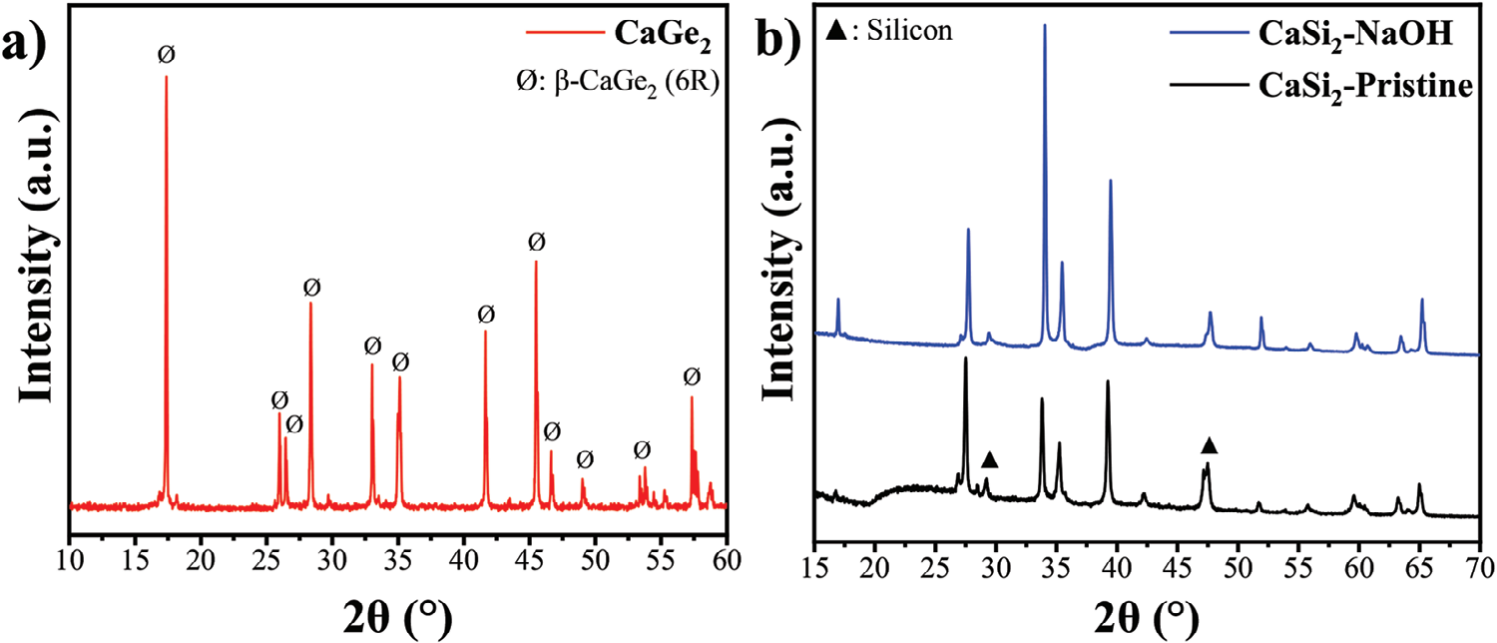
XRD patterns of Zintl phase precursors a) CaGe_2_ and b) CaSi_2_ before (black line) and after (blue line) treatment with NaOH solution to remove impurities.

Understanding the purity of the CaGe_2_ (see methods) and CaSi_2_ precursor materials is crucial for establishing accurate synthesis protocols, particularly when dealing with compounds that exhibit polymorphism. X‐ray diffraction (XRD) analysis of CaGe_2_ (**Figure** **1**a) reveals the presence of the desired pure β‐phase of the Zintl phase, consistent with previous reports.^[^ ^18^, ^21^ ^]^ However, CaGe_2_ can consist of multiple polymorphs, namely 3R, 2H, 4H, and 6R.^[^ ^21^, ^22^ ^]^ The observed absence of the (002) peak, alongside the low intensity of the (003) peak at 16.85°, and the dominant peak at 17.38° corresponding to the (006) plane of the 6R polymorph^[^ ^22b^ ^]^ collectively suggest the prevalence of the 6R polymorph of β‐CaGe_2_, with lattice parameters *a* = 3.99 Å and *c* = 30.645 Å, as the Rietveld refinement analysis also indicates (Figure S2a, Supporting Information).^[^ ^22a^ ^]^ XRD analysis of CaSi_2_ (Figure 1b) indicates the presence of both 6R and 3R polymorphs.^[^ ^22^, ^23^ ^]^ Although the 3R polymorph appears dominant, the Rietveld refinement analysis (Figure S2b, Supporting Information) suggests the coexistence of the 3R and 6R polymorphs and also some bulk‐Si. An aqueous NaOH wash was employed for removal of the impurity XRD peaks corresponding to bulk‐Si at 28.4° and 47.2°. This also produced a slight shift of the XRD peaks to higher angles while maintaining the overall structural integrity, likely due to point defect creation. Treatment of the CaSi_2_ precursor with NaOH proved essential for success of the synthesis, removing Si impurities that would otherwise dilute the acids used for etching, particularly HF.

### 2.2 Germanane (GeH) and Silicane (SiH)

**Figure 2:**
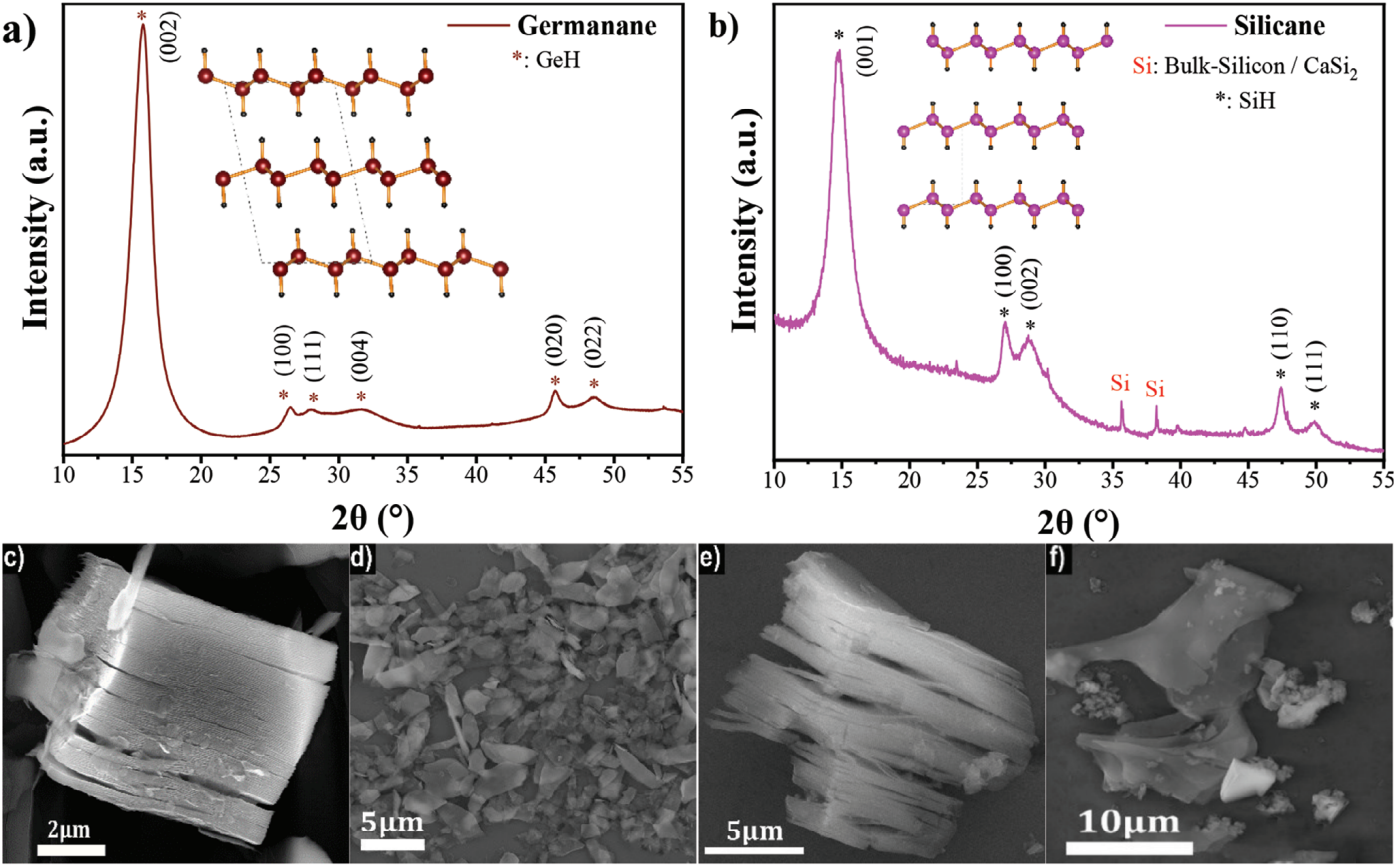
Powder XRD patterns of synthesized Xanes a) GeH (inset: schematic illustration crystal structure of GeH), b) SiH (inset: schematic illustration crystal structure of SiH) and SEM images of c) multilayered GeH, d) multilayered SiH, e) few layered GeH and f) few layered SiH.

XRD analyses of both produced Xanes confirmed the effective removal of Ca interplane atoms using the “in situ” synthesis method. The XRD patterns demonstrate the absence of peaks corresponding to the Zintl phases, with notable shifting of the (002) and (001) peaks to lower angles, indicating an increase in the d‐spacing. In **Figure** **2**a and the case of GeH, the (002) peak occurs at 15.78° with a full width half maximum (FWHM) of ≈1.7°, affirming successful synthesis of sheets of the layered hydrogenated material.^[^ ^17^, ^18^ ^]^ It has been found that the optimum unit cell to define the GeH structure is monoclinic, and refinement of the XRD data gives unit cell parameters of *a* = 6.9 Å, *b* = 3.97 Å, *c* = 11.5 Å and *β* = 102.56°. While literature on GeH synthesis is limited, direct HF approaches exhibit higher peak angles (>17°) and broader FWHM (>2.5°), with similar lattice parameters but a larger β‐angle (105.5°).^[^ ^18^ ^]^ The results here are more similar to GeH synthesized via the HCl approach that gave a β‐angle of 102.18°.^[^ ^17^, ^18^ ^]^

For SiH (Figure 2b), the peaks at 14.68° (001) and at 27.04° (100) correspond to the hexagonal SiH structure with unit cell parameters of *c* = 6.03 Å and *a* = 3.84 Å, in agreement with literature values.^[^ ^4^, ^24^ ^]^ However in previous works, the FWHM is typically very broad and suggests that there are fluctuations in the interlayer distance as well as the number of the layers stacked together.^[^ ^4^, ^24^, ^25^ ^]^ Contrastingly, our method has a FWHM ≈1.5° demonstrating bigger crystallite size and better delamination. Furthermore, the higher intensity of the peaks and the appearance of further crystalline peaks; (002), (110), and (111), further demonstrate effective synthesis of high‐quality crystals compared to previous methods.^[^ ^24b^ ^]^

The scanning electron microscopy (SEM) images presented in Figure 2c,e depicts the morphologies of GeH and SiH respectively, immediately following the etching process. Both materials exhibit an accordion‐like morphology resulting from the removal of Ca from the compact layered precursors (Figure S3, Supporting Information). This morphology is attributed to the uneven and dramatic formation of H_2_ bubbles during the synthesis reaction that drive the layers apart,^[^ ^26^ ^]^ a phenomenon observed in other materials using a similar synthesis approach, such as MXenes.^[^ ^27^ ^]^ The residual forces between layers prevent their complete delamination into individual sheets yet subsequent sonication can effectively disperse these layers, resulting in single or few‐layered flakes.

The SEM images in Figure 2d,f demonstrates the separation of the structures into flakes for GeH and SiH, respectively. The lateral size of these flakes typically ranges from 3 to 20 µm and depends on factors such as the initial lateral size of the precursor, the synthesis approach employed, and any post‐synthesis processing steps.

**Figure 3:**
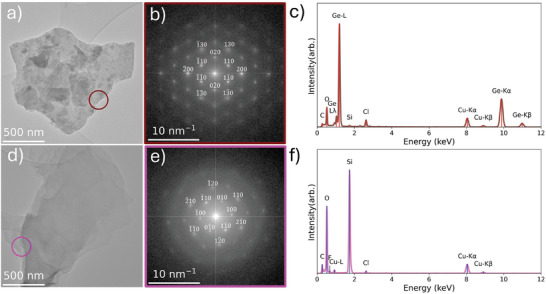
a,d) TEM images, b, d) SAED patterns, and c,f) EDS spectra of flakes from GeH, SiH samples, respectively. Position of SAED aperture used to acquire patterns in b,e) are show by brown and purple circles in a,d), respectively. GeH and SiH STEM EDS spectra acquired from regions shown by insets in Figures S12 and S13 (Supporting Information), respectively.

Transmission electron microscopy (TEM) of the produced flake materials found them to have diameters ranging from 100 nm^−1^ µm as shown in **Figure** **3**a,d. The crystallinity of the produced GeH and SiH was also evaluated in the TEM using selected area electron diffraction (SAED), which was able to identify single crystal regions with bright Bragg peaks, as shown in Figure 3b,e, as well as amorphous regions characterized by diffuse diffraction rings caused by short‐range ordering, as shown in Figure S11 (Supporting Information). From these single crystal regions, the in‐plane lattice parameters of the 2 materials can be established, with SiH *a* = 3.84 ± 0.07 Å, and GeH *a *= 6.63 ± 0.13 Å, *b *= 3.99 ± 0.04 Å, as determined from the SiH second order diffraction spots and GeH 020 and 200 diffraction spots, respectively. These results match well with those derived from XRD spectra, with the exception of the *a* parameter of GeH (6.9 Å). This is likely caused by overlap of peaks arising from diffraction of the {110} planes, which correspond to an enlarged spacing of 6.89 ± 0.13 Å.

The single crystal SAED patterns also contain significant diffuse scattering with hexagonal symmetry that has been previously observed in GeH, but not SiH, and is thought to arise from turbostatic disorder between the layers within the structure.^[^ ^28^ ^]^ The presence of amorphous regions is likely due to degradation of the samples either due to chemical conversion to more stable stoichiometries such as GeO_2_ or SiO_2_, respectively, or due to electron beam induced damage as has been previously observed in GeH.^[^ ^28^ ^]^ To minimize electron beam induced degradation during analysis SAED acquisition was performed with a low electron flux of 76 e‐ nm^−1^.

The elemental composition of the samples was investigated using scanning TEM with energy dispersive X‐ray spectroscopy (STEM EDS), the sum spectra for which are shown in Figure 3c,f. The samples consist of mostly germanane (Ge) or silicon (Si), for GeH and SiH respectively, with carbon (c), oxygen (O), fluorine (F), and chlorine (Cl) also being present in both samples. STEM EDS elemental mapping showed homogenous distributions of elements in both samples (Figures S12 and S13, Supporting Information). These maps show that the majority of the C is related to the carbon film support of the TEM grid. In contrast, Cl, F, and O are mainly associated with the flakes, likely residual surface contamination from the chemical exfoliation of the materials.

**Figure 4:**
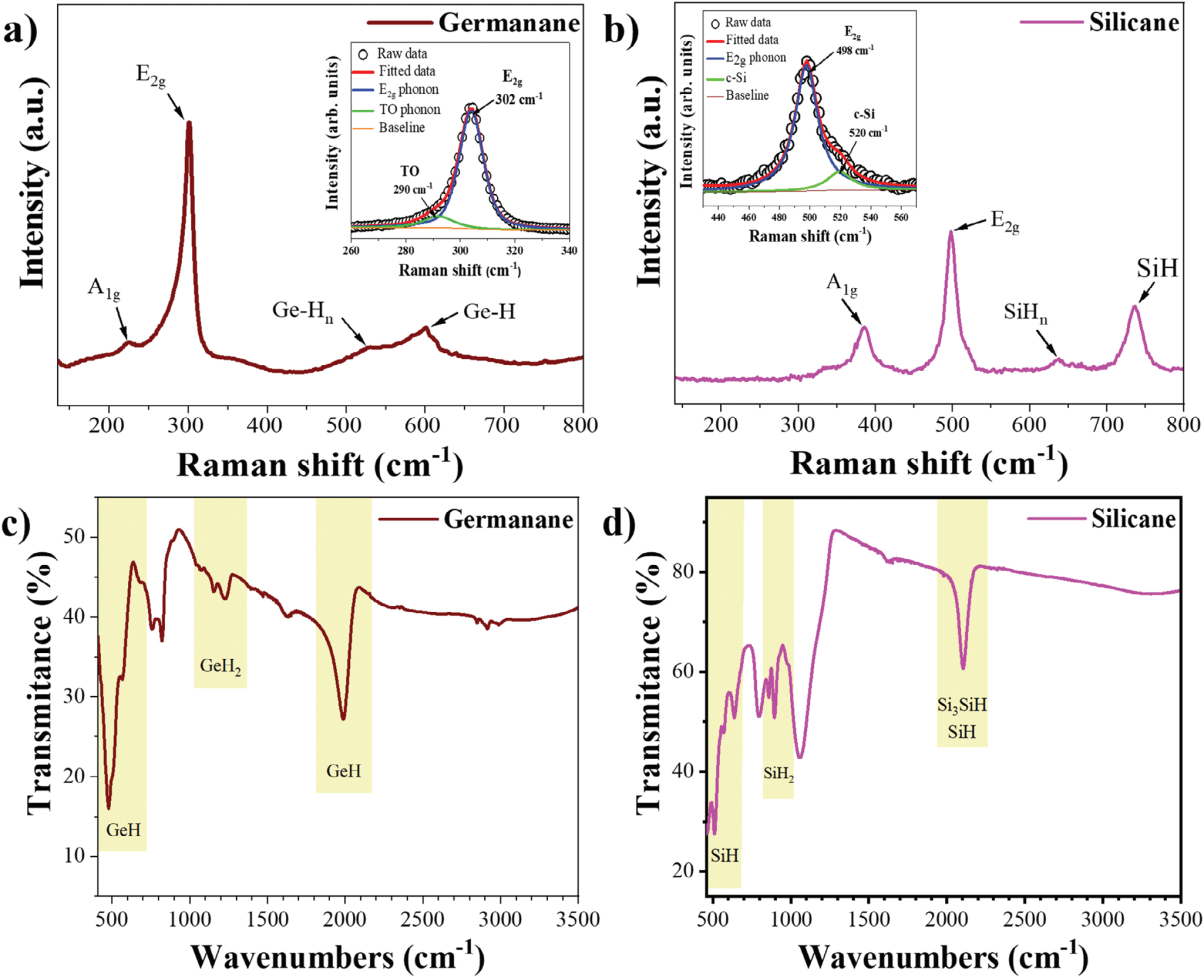
Raman spectrum of synthesized Xanes a) GeH (deconvoluted *E*_2g_ band, inset), b) SiH (deconvoluted *E*_2g_ band, inset), and FT‐IR spectra of c) GeH and d) SiH.

Raman spectroscopy was used to investigate the bonding chemistry of the produced materials. The Raman spectrum of GeH, depicted in **Figure** **4**a, exhibits a characteristic Raman band at 280–320 cm^−1^ attributed to Ge‐Ge stretch (*E*_2g_), consistent with previous reports from both HCl and HF synthesis methods.^[^ ^1^, ^17^, ^18^, ^21^, ^29^ ^]^ Although theory predicts an *A*_1g_ peak at 223 cm^−1^,^[^ ^30^ ^]^ we found this to be slightly shifted to 225 cm^−1^. Again this differs from GeH produced by the HF approach where this peak is absent^[^ ^18^ ^]^ but similar to GeH produced by HCl synthesis.^[^ ^17a^ ^]^ As the *A*_1g_ vibration mode is due to the uniform H‐termination on both sides of the 2D plane this suggests that the milder environment of our synthesis method facilitates more uniform topochemical deintercalation, leading to better kinetics than is common for the HCl‐based synthesis route.^[^ ^30^ ^]^ Our observation of Raman bands at 530–600 cm^−1^ may indicate Ge‐H_n_ (n: 1–3) modes arising from Ge vacancies or edges.^[^ ^21^ ^]^ An alternative explanation suggests these peaks may correspond to transverse optical phonon modes of GeO_2_.^[^ ^31^ ^]^ The corresponding high‐resolution Ge band (See inset in Figure 4a) reveals an asymmetric peak, thus implying the presence of amorphous Ge domains in the sample.^[^ ^32^ ^]^ Further deconvolution of this band unveils a major (intense) peak at ≈302 cm^−1^ associated with the *E*_2g_ phonon in the 2D crystalline germanium phase (i.e. the honeycomb backbone),^[^ ^33^ ^]^ hence this band can be regarded as analogous to the G band for graphene‐based materials.^[^ ^34^ ^]^ Moreover, the deconvoluted Ge band (inset in Figure 4a) revealed another minor (less intense) peak centered at ≈287 cm^−1^ associated with the transverse optical (TO) phonon of the amorphous Ge phase (i.e. disordered domains).^[^ ^35^ ^]^

The Raman spectrum of SiH presented in Figure 4b, shows bands at 320–420, 450–550, 600–680, and 700–770 cm^−1^ characteristic of the Si–Si vibrational modes, in agreement with previous studies^[^ ^24b^ ^]^ confirming the successful etching of CaSi_2_ using the “in situ” HF generation method. The first band at 320–420 cm^−1^ corresponds to *A*_1g_ phonon vibrations of the Si─Si atoms in the 2D plane. The main band at 450–550 cm^−1^ appears asymmetric and further deconvolution of this band reveals two components (see inset in Figure 4b). The first peak centered at ≈498 cm^−1^ corresponds to the E_2g_ phonon arises from the Si─Si bonds in the ring, while the second component, which appears as shoulder in the main band and is centered at ≈520 is attributed to residual crystalline Si.^[^ ^3b^ ^]^ The bands at 600–680 and 700–770 cm^−1^indicate hydrogen termination with Si–H_n_^c^ and Si–H vibration modes, respectively.^[^ ^3b^ ^]^ However, the presence of a shoulder at 341 cm^−1^ suggests some degradation of SiH, possibly indicating Si–O vibration modes.^[^ ^36^ ^]^

Fourier‐transform infrared spectroscopy (FT‐IR) analysis was used to further study the hydrogen terminations within the materials. FT‐IR of GeH (Figure 4c) shows peaks at 478 and 566 cm^−1^ corresponding to Ge‐H wagging (*B*_3u_), while those at 759 and 822 cm^−1^ signify H‐Ge‐H bending (*A*_1_), potentially arising from Ge vacancies or edge effects.^[^ ^21^ ^]^ These could further support for the interpretation of the 386 and 498 cm^−1^ bands in Raman analysis being produced by Ge–H_n_ modes. Notably, the peak at 1987 cm^−1^ corresponding to the Ge–H stretch mode deviates from typical literature values (≈2000 cm^−1^),^[^ ^17^, ^18^, ^29^ ^]^ hinting at a combination of 2H and 6R structures for the final 2D material.^[^ ^21^ ^]^ The broad and asymmetric nature of the 1987 cm^−1^ peak suggests the presence of both mono‐ and dihydride species, with the former prevailing.^[^ ^37^ ^]^

Similarly, the FT‐IR spectrum for SiH (Figure 4d) confirms further the synthesis of silicon nanosheets, a departure from conventional approaches.^[^ ^24^, ^26^, ^38^ ^]^ Peaks at 513 cm^−1^ (Si–Si plane stretching), 638 cm^−1^ (Si–H bending), 861 cm^−1^ (SiH_2_), and 2104 cm^−1^ (Si_3_SiH/SiH) indicate SiH formation, while the peak at 895 cm^−1^ (SiH_2_/SiOH_c_) suggests the potential presence of hydroxyl or hydrogen at edges and/or defect sites.^[^ ^24b^ ^]^ Oxidation reactions are evident with the 1056 cm^−1^ peak representing Si─O─Si asymmetric stretching, indicating oxygen atom incorporation with Si atoms. The pre‐treatment of the CaSi_2_ precursor with NaOH results in reduced transmission intensity for the Si─O─Si peak at 1056 cm^−1^, suggesting a lower degradation rate and nearly complete removal of carbon contaminations (1380–1394 cm^−1^: C─H, and 2899–2989 cm^−1^: CH_x_) (Figure S4, Supporting Information). In comparison to traditional HCl synthesis approaches the Si–O–Si peaks at 1016 and 795 cm^−1^ are significantly less intense, while the 2097 cm^−1^ Si_3_SiH/SiH peak is more intense in our “in situ” method.^[^ ^3^, ^24^, ^25^, ^36^, ^39^ ^]^ Additionally, the lack of peaks at 3410 cm^−1^ (Si─OH) and 2240–2350 cm^−1^ (O_3_SiH) indicate that we do not have Kautsky‐type siloxene as has been reported in other works.^[^ ^3^, ^24^, ^39^, ^40^ ^]^

**Figure 5:**
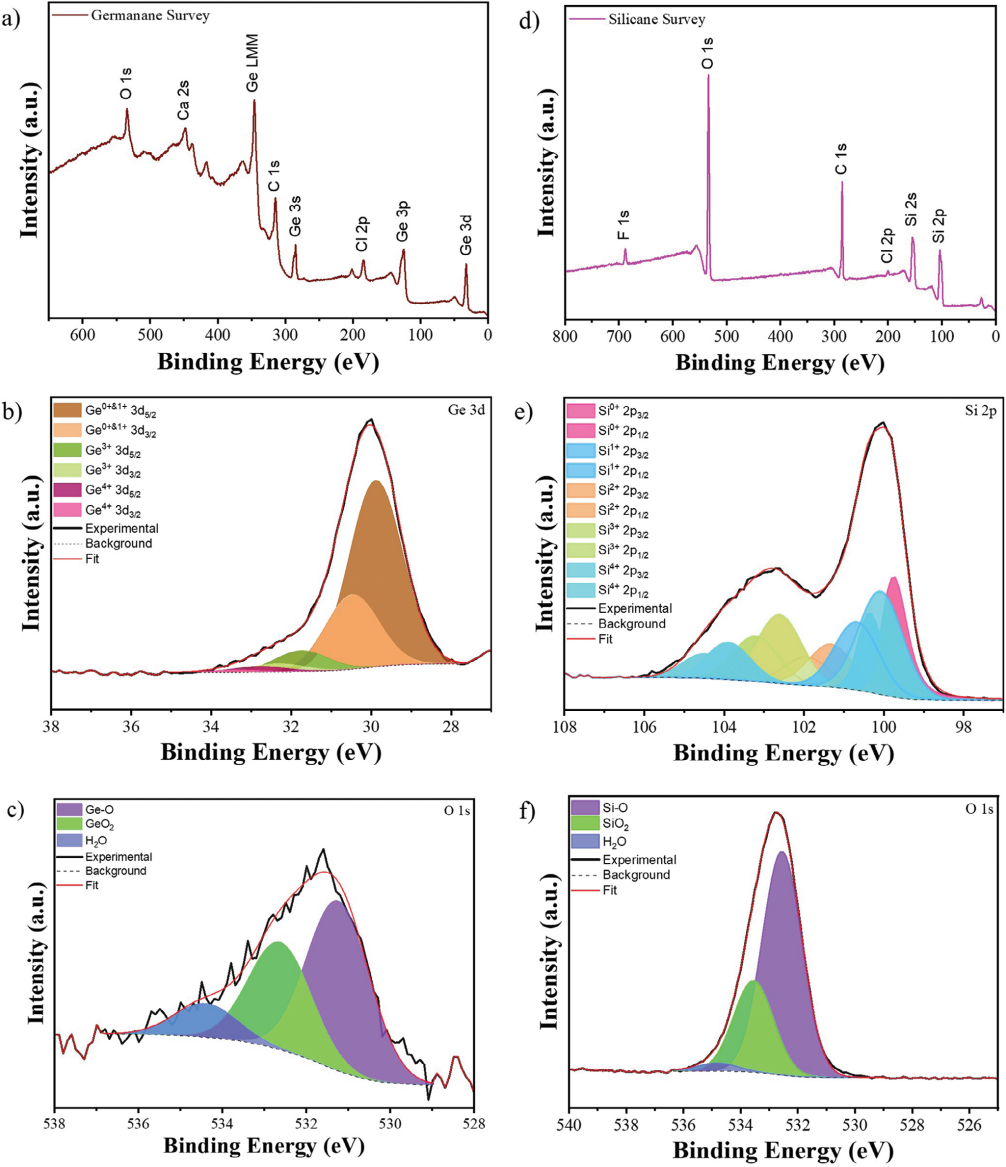
XPS of GeH and SiH. a) GeH survey scan with high‐resolutions scans of b) Ge 3d, c) O 1 s, and d) SiH survey scan with high‐resolutions scans of e) Si 2p and f) O 1 s.

X‐ray photoelectron spectroscopy (XPS) was used to further investigate the elemental composition of the produced Xanes. All spectra were calibrated to adventitious carbon, set to a binding energy (BE) of 284.8 eV. The survey spectra (**Figure** **5**a,d) demonstrate the presence of O, C, F, and Cl for both materials, and silicon (Si) or germanium (Ge) for the corresponding SiH and GeH, respectively, consistent with STEM EDS measurements. The composition of the GeH sample indicates an atomic ratio of Ge:O:Cl to be 70:21:9, while for the corresponding SiH sample, the atomic ratio of Si:O:Cl:F is 59:37:0.5:3.5 also in agreement with STEM EDS. The high‐resolution spectrum of the 3d Ge region in Figure 5b reveals three pairs of peaks (0.59 eV split). The Ge main peak associated with GeH is observed at 29.8 eV/30.4 eV, a combination of Ge^0+^ at slightly higher BE than elemental Ge,^[^ ^41^ ^]^ attributed to more electronegative Ge‐H terminations, and Ge^1+^ for potential other terminations Ge_3_‐Ge‐T_x_ (T_x_: O, F, Cl, and H_2‐3_).^[^ ^42^ ^]^ The other two minor peaks are attributed to higher oxidation states of germanium, Ge^3+^ at 31.7 eV/32.3 eV (GeO_x_/Ge–O) and Ge^4+^ at 32.8 eV/ 33.4 eV (GeO_2_), respectively, due to oxygen defects into GeH (edges and/or defect sites) and surface oxidation, similar to that observed in germanium nanosheets.^[^ ^4a^ ^]^ The O high‐resolution spectrum (Figure 5c) confirms further the inevitable partial oxidation of GeH, where the 532.9 eV peak is connected to the oxygen species of GeO_2_, while the 531.4 eV peak could indicate Ge–O species at the edges and/or the defect sites of the GeH. The ≈534 eV peak corresponds to adsorbed water. Considering the O atomic ratio in relation to possible bonds with other elements (GeO_2_, Ge‐O, C─OH/C─O─C, etc.), the calculated O content in GeH is negligible (<1.5%). Additionally, the high‐resolution spectrum for the Cl 2p orbit (Figure S5a, Supporting Information) shows two doublets with a 1.55 eV splitting, with the first peak at 197.6 eV/199.2 eV likely connected to unwashed CaCl_2_ salt and the second Cl peak at 198.9 eV/200.5 eV more likely connected to adsorbed Cl^−^ on the GeH.^[^ ^43^ ^]^ The adsorbed Cl content is calculated to be 3%.^[^ ^44^ ^]^

In SiH, the Si high‐resolution spectrum (Figure 5e) is more complex and has been deconvoluted into five doublets (split 0.6 eV), with the 99.8 eV/100.4 eV (Si^+0^) and 100.1 eV/100.7 eV (Si^+1^) are related with SiH, with similar chemistry as mentioned for GeH, with Si–Si‐H and Si_3_–Si‐T_x_, respectively.^[^ ^24a,b^ ^]^ The peaks at 101.3 eV/101.9 eV (Si^2+^: SiO_x_/Si–O), 102.6 eV/103.2 eV (Si^3+^: Si–O–Si), and 103.9 eV/‐104.5 eV (Si^4+^: SiO2) are related to oxidation traces of SiH and O interatoms in the silicon 2D lattice.^[^ ^24^, ^36^, ^40^, ^45^ ^]^ A previous report using concentrated HF for additional etching after conventional synthesis with HCl showed that the additional etching step minimizes the intensity of Si^+2^ and Si^+4^ peaks.^[^ ^2b^ ^]^ The oxygen atomic ratio for SiH is significantly higher than for GeH, but with similar behavior. In the SiH high‐resolution spectrum (Figure 5f), three peaks slightly shifted to higher BE compared to the same spectrum of GeH are observed, due to more electronegative Si, centered at 532.6, 533.5, and 534.8, assigned to SiO_x_/Si–O–Si, SiO_2_, and H_2_O, respectively. By considering the O atomic ratio in relation to possible bonds with other elements as done with GeH analysis (SiO_2_, Si‐O, C─OH/C─O─C, etc.), the calculated O content in SiH is 14% (mainly Si─O─Si). Finally, the Cl spectrum (Figure S5b, Supporting Information) of SiH shows only one pair of peaks, with Cl 2p_3/2_ centered at 199.6 eV and corresponding to Cl ions. However, F is present at slightly higher quantities than Cl (Figure S5c, Supporting Information) with the adsorbed F and Cl ions quantified to be 1.5% and <0.4%, respectively.

**Figure 6:**
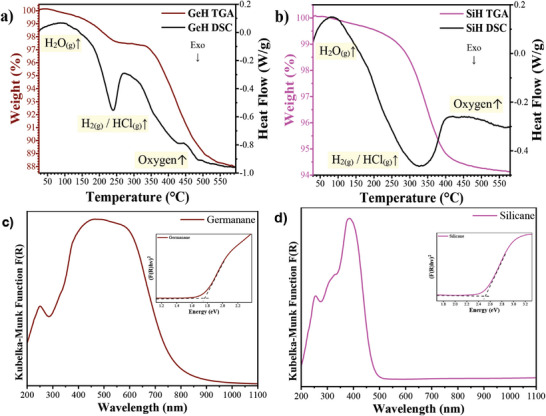
TGA and DSC of synthesized Xanes a) GeH (black line DSC & brown line TGA) and b) SiH (black line DSC & purple line TGA). Diffuse reflectance absorbance (DRA) spectrum plotted as Kubelka‐Munk function versus wavelength; inset: Tauc‐plot analysis of the Kubelka‐Munk function of c) GeH in propanol and d) SiH in ethanol.

The thermal stability of both GeH and SiH materials was investigated through thermogravimetric analysis (TGA) and differential scanning calorimetry (DSC) under an inert atmosphere, as depicted in **Figure** **6**. GeH TGA (Figure 6a) reveals two distinct steps of mass loss. The initial step, accounting for a mass loss of 2.5% at 280–290 °C, is accompanied by an endothermic peak in the DSC ≈130 °C, indicative of entrapped water removal. Concurrently, dehydrogenation occurs in the same temperature range, although not clearly discernible in the DSC. Theoretically, complete dehydration of fully hydrogen‐terminated germanane should result in a maximum weight loss of ≈1.35%, suggesting the presence of residual water. Notably, the temperature of the first plateau exceeds previously reported measurements (200–250 °C),^[^ ^17^, ^46^ ^]^ potentially indicating a more uniform and smaller defect density of the final material. Subsequently, a mass loss of ≈9% occurs at 545–555 °C, accompanied by an exothermic peak ≈240 °C in the DSC, attributed to the loss of HCl_(g)_,^[^ ^17^, ^46^ ^]^ along with two endothermic peaks ≈290 and ≈450 °C indicative of GeH decomposition and/or removal of other terminations (‐O/‐OH).

Similarly, SiH thermal analysis in Figure 6b reveals two distinct weight loss steps. The initial step at 160 °C involves a minor weight loss of ≈0.3% attributed to water evaporation, while the subsequent step at 445 °C demonstrates a more significant weight loss of 5.2%, attributed to hydrogen loss and Cl removal, as previously reported.^[^ ^24b^ ^]^ The theoretical weight loss of all hydrogens for infinite SiH is ≈3.4%. DSC analysis reveals exothermic loss of H and Cl, manifested as a broad peak ≈105–325 °C. The origin of HCl may be through condensation reactions involving ^*^SiCl/^*^GeCl and ^*^SiH/^*^GeH or from intercalated HCl.^[^ ^24b^ ^]^

We now consider the photon and environmental stability of the synthesized SiH and GeH materials. SiH appears to have significant sensitivity to light, as evidenced by degradation and discoloration to a white‐grey hue when stored in a clear transparent vial under vacuum for 45 days. On the other hand, samples stored in dark environments with no light exposure for 45 days retained their strong yellow coloration. Nonetheless, SiH does appear to have mild sensitivity to air in ambient conditions with slight discoloration observed in the sample stored in an open atmosphere for 45 days, (Figure S6, Supporting Information). This sensitivity to light could explain the higher concentration of oxygen that showed in XPS and STEM EDS, as oxygen introduced in the lattice by either substituting Si atoms, cluster adsorption, or by placing oxygen atoms in random positions as degrade over time.^[^ ^47^ ^]^ GeH, was found be more stable in ambient conditions with no change in the appearance or XRD pattern regardless the light exposure after 45 days (Figure S7, Supporting Information). However, a more comprehensive analysis is necessary to further elucidate the kinetics and mechanisms underlying this degradation phenomenon.

Both GeH and SiH present promising prospects for semiconductor applications owing to their small bandgaps (*E*_g_). The UV–vis diffuse reflectance spectra of GeH and SiH were analyzed using Kubelka–Munk functions plotted against wavelength (Figure 6c,d), and subsequently subjected to Tauc plot analysis to determine their respective *E*_g_ values (Figure 6c–d insert). The direct *E*_g_ of GeH was found to be 1.75 eV, higher than GeH synthesized with HF (1.4 eV),^[^ ^4^, ^18^ ^]^ but within the range of GeH materials synthesized with concentrated HCl approach (1.52–1.81 eV).^[^ ^1^, ^4^, ^17^, ^21^, ^46^ ^]^ SiH due to its close indirect energy proximity to a direct transition, past empirical findings has been reported as both a direct and an indirect bandgap material.^[^ ^4^, ^24^ ^]^ We find an indirect E_g_ of 2.47 eV similar to previous studies (≈2.48 eV).^[^ ^4^, ^24^ ^]^ The bandgap of GeH and SiH can vary due to factors such as the number of layers in the unit cell, stacking arrangement, the size of the flakes, and the interlayer distance within the crystal.^[^ ^2^, ^18^, ^24^, ^48^ ^]^ The large bandgaps we observe are indicative of high‐quality crystals as even partial substitution of hydrogen with hydroxyl, chlorine or fluorine has been predicted to decrease *E*_g_ values.^[^ ^1^, ^49^ ^]^

The use of Xanes to produce flexible electronics or printed semiconductors requires the materials be dispersible in a suitable solvent. Water is found to be a poor solvent for both Xanes (Figures S9 and S10, Supporting Information), prompting solubility trials to identify more suitable solvents. A range of organic solvents were assessed (Tables S1 and S2, Supporting Information) over a 3‐day evaluation period. Figures S9 and S10 (Supporting Information) illustrate the solubility tests for GeH and SiH, respectively. Isopropanol emerged as the most effective solvent for dispersing GeH among the options tested. For SiH, a wider range of solvents was tested due to the capacity to synthesize larger quantities. However, none of these solvents proved to be effective for dispersing the silicon‐based nanomaterial. Hexane demonstrated the best dispersion after 3 days, followed by a range of polar aprotic solvents (e.g., 1‐Methyl‐2‐pyrrolidinone, dimethyl sulfoxide, *N,N*‐Dimethylformamide, etc.).

## 3 Conclusion

This work has demonstrated the successful synthesis of GeH and SiH crystal flakes using a new “in situ” approach involving a halogenated (fluoride) salt dissolved in an aqueous acidic solution reducing reaction times to just 15 min. XRD and TEM revealed high crystalline quality with minimal impurities and relatively large crystallite sizes. Analysis of reaction product through STEM‐EDS, FT‐IR, Raman, and XPS confirmed the presence of surface moieties such as –O(H), –F, and –Cl. The former species is attributed to edge and defect sites whilst the latter two are expected to be associated with adsorbed surface impurities. Deconvolution of the Raman *E*_2g_ phonon mode for germanane and observation of small contribution of TO phonons of the amorphous Ge phase indicates a low defect, high crystallinity material. Similarly, silicane appears to have a low defect concentration but the presence of the Si‐O vibrational mode is observed, likely due to the presence of moisture and oxygen. These promising results demonstrate this approach as a fast and safer method for synthesizing large flakes of 2D semiconducting Xane materials from Zintl phases compared to previous routes, for applications in flexible electronics, optoelectronics, and energy storage. Whilst further kinetic and thermodynamic studies are necessary to understand degradation behavior, as well as a detailed analysis of defect states and the influence of surface terminations on band energies, this relatively niche and emerging class of materials has promise for applications in opto‐/micro‐electronics and catalysis, which the developed synthesis method will facilitate.

## 4 Experimental Section

### Precursors

In a typical batch, calcium (Ca) and Ge powder were mixed in a ratio of 1:1.75. This mixture was then placed in a cylindrical lidded alumina crucible, enclosed in an evacuated fused quartz tube. The mixing of the two metals and the crucible filling were conducted in a glove box under inert atmosphere (Nitrogen). Subsequently, the sealed quartz tube was placed in a furnace, with steps: 1) heating to 1025 °C at a rate of 8.3 °C min^−1^, 2) annealing at 1025 °C for 20 h, 3) slow cooling to 500 °C at a rate of 0.1 °C min^−1^ and finally, 4) cooling down to room temperature at a rate of 0.2 °C min^−1^. The final product is β‐CaGe_2_ crystal (Figure S1, Supporting Information) with metallic appearance (moisture sensitive due to Ca). On the other hand, the precursor of SiH could easily be purchased in high purity (Figure S1, Supporting Information). However, both precursors could crystalize in a variety of polymorphs.^[^ ^21^, ^22^, ^23^ ^]^

### Reagents

Hydrochloric acid (Aq. 37 wt.%, laboratory reagent grade) was purchased from Fischer Scientific. Lithium fluoride (≥98.5%, 325 mesh) purchased from Thermo Scientific. Deionized (DI) water used was purified with Mili‐Q EQ7008, Merck (resistivity: 18.2 MΩ cm^−1^). CaSi_2_ (technical grade) was purchased from Sigma–Aldrich. Calcium (granular, purity ≥99%), Germanium (powder, ≥99.999%, 100 mesh) were purchased from Sigma–Aldrich. Methanol (HPLC, gradient grade) purchased from Acros Organics. Isopropanol (≥99.8%, analytical reagent grade) was purchased from Fisher Scientific. Acetonitrile (≥99.9%, HPLC plus) purchased from Sigma–Aldrich. Ammonia in methanol (7 N) purchased from Acros Organics. Ethanol absolute (≥99.97%) purchased from VWR Chemicals.

### Synthesis Protocols—GeH Main Synthesis

To prepare the liquid etchant, 40 mL of hydrochloric acid (HCl) with a 12 m concentration was poured into an appropriate reaction vessel, such as a PTFE vessel compatible with hydrofluoric acid (HF). Subsequently, 6 g of lithium fluoride (LiF) was added, and the mixture was stirred for 15 min to ensure thorough dissolution of the salt in the acid. 1 g β‐CaGe_2_, was gradually added to the etchant solution at intervals of 2 min between additions to prevent rapid temperature increase. The reaction mixture was vigorously stirred at 30 °C for 18 h. It was important to note that hydrogen gas was generated during the reaction, which may carry hazardous vapor byproducts such as HF and HCl. Proper ventilation must be employed to mitigate potential risks. Observation of a maroon coloration within the reaction vessel was expected over time, with finer β‐CaGe_2_ crystals leading to a more rapid appearance of this color. After 18 h, the acidic mixture was transferred to a 250 mL centrifuge bottle compatible with HF, where it underwent washing via centrifugation with 0.5 m HCl to remove reaction byproducts such as CaF_2_ and LiF. This washing process was repeated several times until no foreign bodies were visible in the mixture. After each centrifugation cycle (at 10 000 rpm for 20 min), the supernatant was discarded, and fresh 0.5 m HCl or DI water was added for the next cycle. Subsequent washes with deionized (DI) water were performed for several cycles to neutralize the pH to ≈7. The resulting GeH slurry was poured into a petri dish and subjected to agitation with DI water and/or isopropanol to disperse any agglomerations. The slurry was then dried overnight (for 16 h) in a vacuum oven at 45 °C. Finally, the dried fine maroon powder was collected and stored in an opaque container under vacuum within a desiccator for further use.

### Synthesis protocols—CaSi_2_ NaOH Treatment and SiH Main Synthesis

For the pre‐synthesis of SiH, initial washing of CaSi_2_ was conducted to eliminate any impurities. 5 g of CaSi_2_ was introduced into a 250 mL solution of 1 m NaOH and stirred at room temperature overnight (18 h), preferably with inert gas bubbling to minimize degradation during the pre‐treatment. Subsequently, after the 18 h period, the solution was divided into two 250 mL centrifuge bottles for washing with deionized (DI) water through several cycles to achieve a near‐neutral pH of ≈8. Following each wash cycle, the alkaline solution was centrifuged at 10 000 rpm for 30 min, with the supernatant discarded and fresh DI water added for the subsequent cycle. The washed CaSi_2_ was then transferred to a petri dish and agitated with DI water to remove any agglomerations before being dried overnight (18 h) in a vacuum oven at 55 °C. The resulting black powder of the washed CaSi_2_ was collected and deemed ready for the main synthesis process.

The main synthesis process began with the preparation of the liquid etchant. 100 mL of hydrochloric acid (HCl) with a concentration of 12 m was poured into a suitable reaction vessel, preferably made of HF‐compatible material such as PTFE. Subsequently, 1.5 g of lithium fluoride (LiF) was added, and the mixture was stirred for 15 min to ensure proper dissolution of the salt in the acid. The reaction vessel containing the “in situ” HF mixture was then placed in an ice bath, and the acidic mixture was continuously stirred until it stabilized at the temperature of the ice bath.

*Warning*: The reaction between CaSi_2_ and the acidic mixture was extremely exothermic and the mixture should preferably be at subzero temperatures during the introduction of the Zintl phase into the reaction mixture, with addition carried out in very low doses.

Gradually and cautiously, 1 g of washed‐CaSi_2_ was added to the mixture at intervals of 5 to 10 min to prevent rapid temperature increases. It was important to note that effervescence creating a foam may occur on the surface of the mixture, increasing the volume of the reaction. Therefore, a 500 mL reaction vessel and vigorous stirring were recommended to ensure proper mixing of the washed‐CaSi_2_ powder with the acidic mixture. The mixture was vigorously stirred at 0 °C (ice bath) for up to 1 h. Throughout the reaction, hydrogen gas was generated, potentially carrying hazardous vapor byproducts such as HF and HCl, necessitating proper ventilation. A strong yellow coloration in the reaction vessel was expected after several minutes into the reaction. After the reaction, the acidic mixture was transferred to a 250 mL centrifuge bottle made of HF‐compatible material, such as PP, and washed with 0.5 m HCl to remove reaction byproducts such as CaF_2_ and LiF. This washing process was repeated several times until no foreign bodies were visible in the mixture. Subsequent washes with deionized (DI) water were performed to neutralize the pH to ≈7. After each centrifugation cycle (at 10 000 rpm for 30 min), the acidic supernatant was discarded, and fresh HCl or DI water was added for the next cycle. The resulting SiH slurry was poured into a petri dish and agitated with DI water and ethanol to remove any agglomerations before being dried overnight (18 h) in a vacuum oven at 45 °C. Finally, the fine yellow powder was collected and stored in an opaque container under vacuum within a desiccator for further use.

### Characterization

XRD: XRD patterns were collected with Philips X'pert Pro MPD with a Cu source (Cu K_α_, *λ* = 1.54 Å). All the samples were powders compressed on zero‐background substrates under ambient atmosphere and minimal lighting. Data acquisition: 12–16 h, range 5–70° 2Θ and step size 0.012°.

Raman spectroscopy: Raman spectra and optical microscopy images were collected with a Renishaw in via Raman microscope. Samples were drop‐casted on Si/SiO_2_ substrate with DI water as solvent for uniformity. 532 nm (Kimmon Koha IK Series He–Cd) and 785 nm (Renishaw HPNIR) lasers were used with grating 1200 grooves mm^−1^ (532 nm) and 2400 grooves mm^−1^ (785 nm). The exposure times was 15 s for multiple acquisitions.

Fourier Transformation – Infrared Spectroscopy: FT‐IR spectra were collected with Thermo Scientific Nicolet iS50 FT‐IR spectrometer and the smart iTX attenuated total reflectance accessory.

Transmission electron microscope (TEM) imaging and SAED was performed using a Thermofisher Talos TEM operated at 200 kV with a 0.21 ± 0.1 µm SAED aperture. SAED patterns were recorded using a Quantum Detectors Merlin Quad detector with 512 × 512 pixels. The cross visible in the diffraction data was a result of the missing pixels at the join between the Merlin detector's 4 chips. STEM‐energy dispersive X‐ray spectroscopy (EDS) data was acquired using the Talos’ Super‐X EDS detector system (4 detectors with a total collection solid angle ≈0.9 srad).

Scanning electron microscopy: SEM images and energy dispersive X‐ray spectroscopy analysis (EDS) was collected with a Tescan Mira3 SEM at 20 kV and 10A beam intensity and Oxford Instruments, INCAx‐act EDS sensor. Samples were drop‐casted on Si/SiO_2_ substrate with DI water and alcohol as solvent after bath sonication (37/80Hz) and attached to aluminum studs with carbon stickers.

Thermogravimetric analysis & Differential scanning calorimetry: TGA & DSC measurements took place with an STA 449 F3 Jupiter thermal analyzer (NETZSCH) and the samples were powders in an alumina (Al_2_O_3_) crucible. The argon flow rate was 50 mL min^−1^ and a ramp rate of 10 °C min^−1^.

X‐ray photoelectron spectroscopy: XPS measurements were collected with monochromatized Al_Ka_ source and pass energy was 80 and 20 eV for survey and core level scans, respectively. Samples were prepared as pressed powders.

UV–vis spectroscopy: UV–vis performed in a Perkin Elmer, Lambda 365 UV–vis spectrometer. The samples were measured as powders in an integrated sphere.

Solubility test: ≈1 mg of SiH and GeH were added in 1 mL of solvent, then sonicated in a bath sonicator (80 Hz) and finally shaken by hand. All the vials were kept under minimal lighting.

## Conflict of Interest

The authors declare no conflict of interest.

## Supporting information



Supporting Information

## Data Availability

The data that support the findings of this study are available from the corresponding author upon reasonable request.
