# Supplementary material for: Faster and Safer “In situ” Synthesis of Germanane and Silicane
Source: Small Methods. 2024 Oct 12;9(3):2400964. doi: 10.1002/smtd.202400964 (PMC11926497; doi:10.1002/smtd.202400964)

Supporting Information

**Faster and Safer “In-situ” Synthesis of Germanane and Silicane**

*Yiannis Georgantas^*^, Theodosis Giousis, Francis P. Moissinac, Gareth R. Tainton, Sarah J. Haigh, Mark A. Bissett^*^*

Yiannis Georgantas, Francis P. Moissinac, Gareth R. Tainton and Mark A. Bissett
Department of Materials, Henry Royce Institute, National Graphene Institute, University of Manchester, Ox-ford Road, Manchester M139PL, U.K.

Theodosis Giousis

Department of Materials Science & Engineering, University of Ioannina, 45110, Ioannina, Greece; Zernike Institute for Advanced Materials, University of Groningen, Nijenborgh 4, 9747 AG Groningen, the Netherlands

** Corresponding authors: Yiannis.Georgantas@manchester.ac.uk and Mark.Bissett@manchester.ac.uk*

**Contents**

**Figures:**

**Figure S1.** Photograph of Zintl phases as precursors: a) CaGe_2_ crystals and b) CaSi_2_ powder.

**Figure S2.** Rietveld refinement of the XRD data of stoichiometric a) CaGe_2_ and b) CaSi_2_ sample. A mixture of 6R and 3R polymorphs was employed as the structural model and additional cubic silicon for the CaSi_2_.

**Figure S3.** SEM images of a) CaGe_2_ crystals, b) CaSi_2_ powder pristine and e) CaSi_2_ powder after NaOH treatment.

**Figure S4**. FT-IR spectra of SiH with NaOH treatment of CaSi_2_ (purple line) and without (Black line).

**Figure S5.** X-Ray photoelectron spectroscopy (XPS) high-resolutions scans of GeH a) Cl 2p and SiH b) Cl 2p and c) F 1s.

**Figure S6.** SiH samples stored (for 45 days) in various conditions.

**Figure S7.** XRD of GeH comparing a fresh synthesised batch and the material after 10 days and 45 days storage in ambient conditions.

**Figure S8**: XRD Patterns illustrating the optimization of SiH synthesis for a) the effect of reaction time (blue line: 15’, green line: 45’, black line: 1h30’ and red line: 14h), b) the use of different fluoride salts (red line: NaF & black line: LiF), c) HCl molarity (black line: 6 M, red line: 9 M & blue line: 12 M) and d) the importance of NaOH treatment of CaSi2 (red line: untreated & black lined: NaOH treated).

**Figure S9.** Solubility test of GeH in a variety of solvent for 3 days.

**Figure S10.** Solubility test of SiH in a variety of solvents for 3 days under minimal light exposure.

**Figure S11.** TEM images and the corresponding SAED patterns of amorphous areas of flakes of (a, b) GeH and (c,d) SiH. The circles in a and c indicate the location of the SAED analysis for both flakes.

**Figure S12.** STEM EDS elemental maps of GeH flake. Red inset shows region from which the EDS sum spectrum shown in Figure 3c was acquired.

**Figure S13.** STEM EDS elemental maps of a SiH flake. Red inset shows region from which the EDS sum spectrum shown in Figure 3f was acquired.

**Figure S14.** TEM images of GeH flake region from which the crystalline SAED pattern shown in Figure 3(b) was acquired, with the inset FFT of the final image to display the lack of periodic details that could be used to determine details about the crystal structure.

**Notes:**

**Note 1.** Synthesis optimization procedure

**Note 2.** Reagents supplementary (Figure S6-S8)

**Tables:**

**Table S1.** The solvents that used for GeH solubility test.

**Table S2.** The solvents that used for SiH solubility test.

**Figure S2.** Rietveld refinement of the XRD data of stoichiometric a) CaGe_2_ and b) CaSi_2_ sample. A mixture of 6R and 3R polymorphs was employed as the structural model and additional cubic silicon for the CaSi_2_.

**a)**


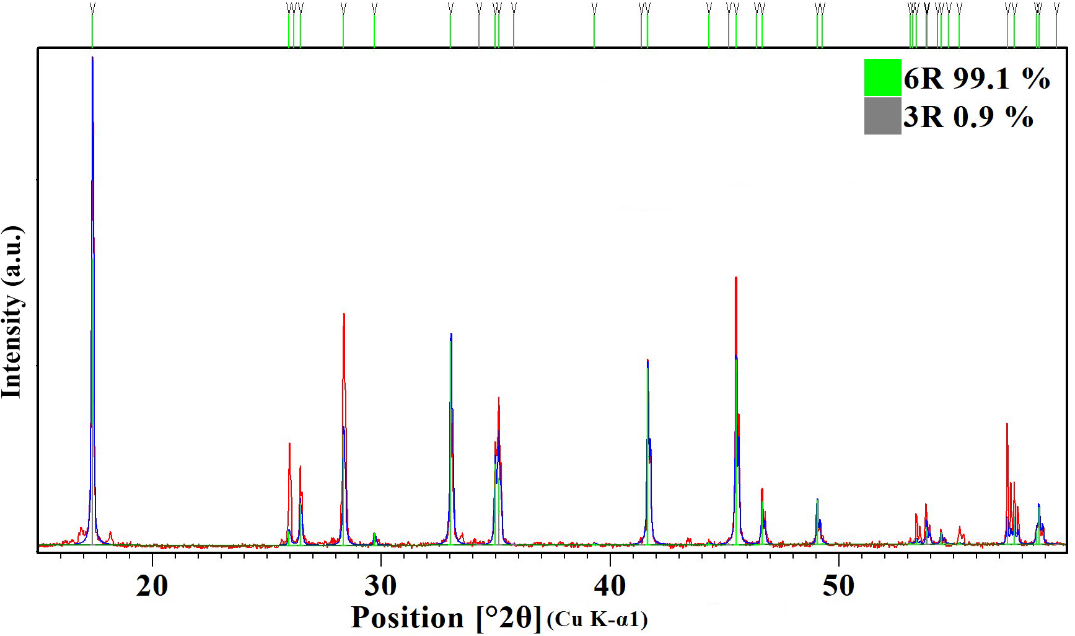


**CaGe_2_**


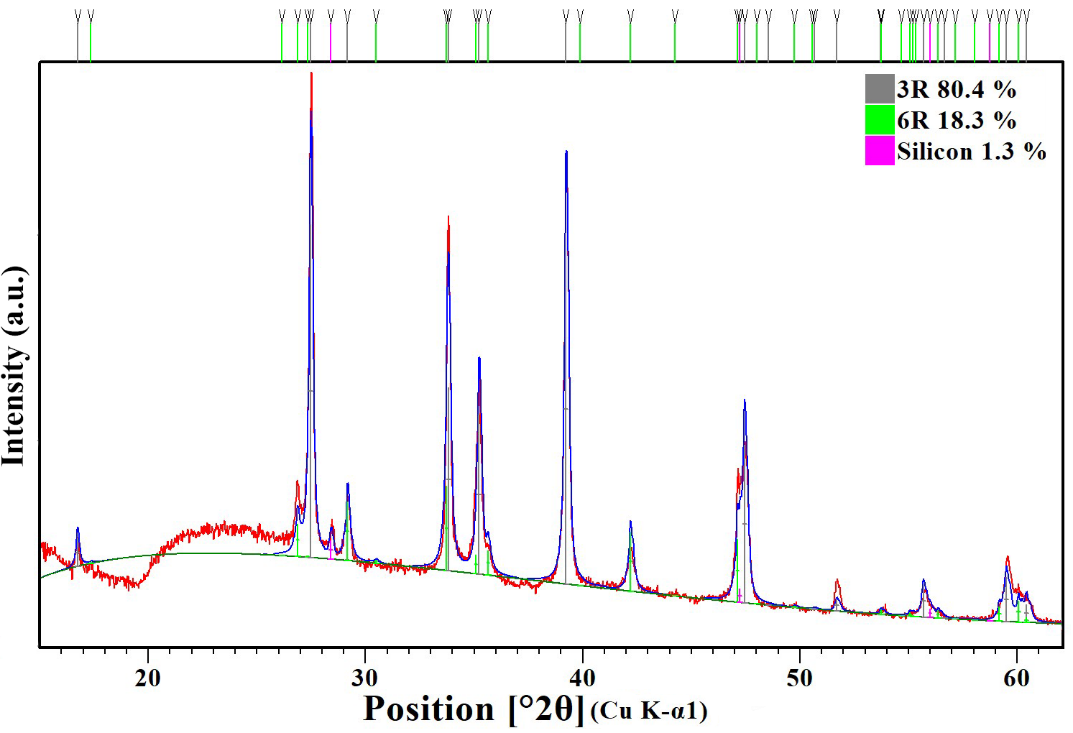


**CaSi_2_**

**b)**


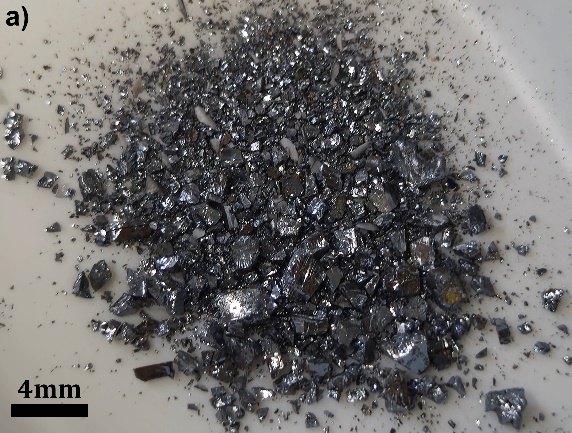

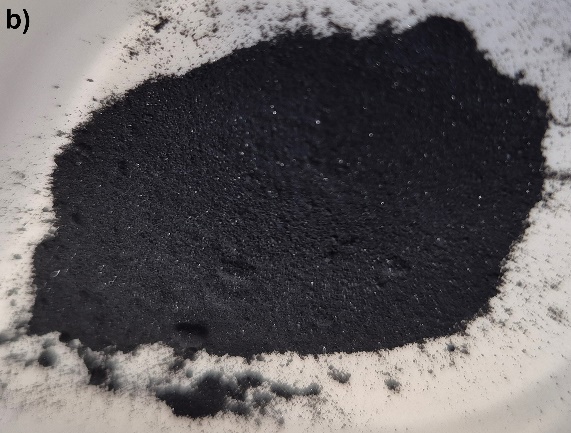


**Figure S1.** Photograph of Zintl phases as precursors: a) CaGe_2_ crystals and b) CaSi_2_ powder.

**Figure S3**. SEM images of a) CaGe_2_ crystals, b) CaSi_2_ powder pre-treatment and e) CaSi_2_ powder after NaOH treatment.


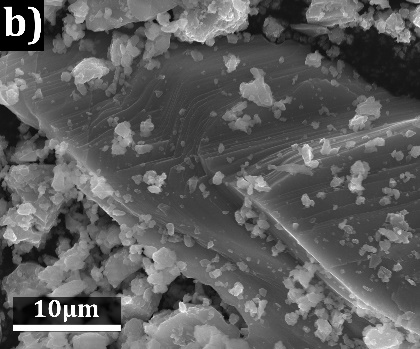

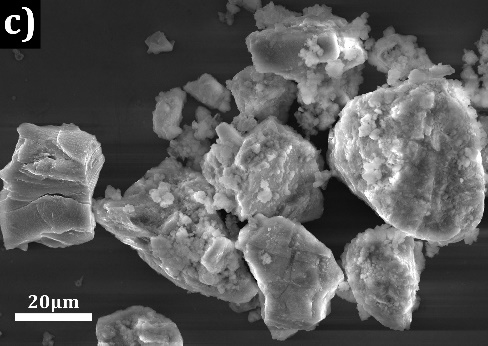

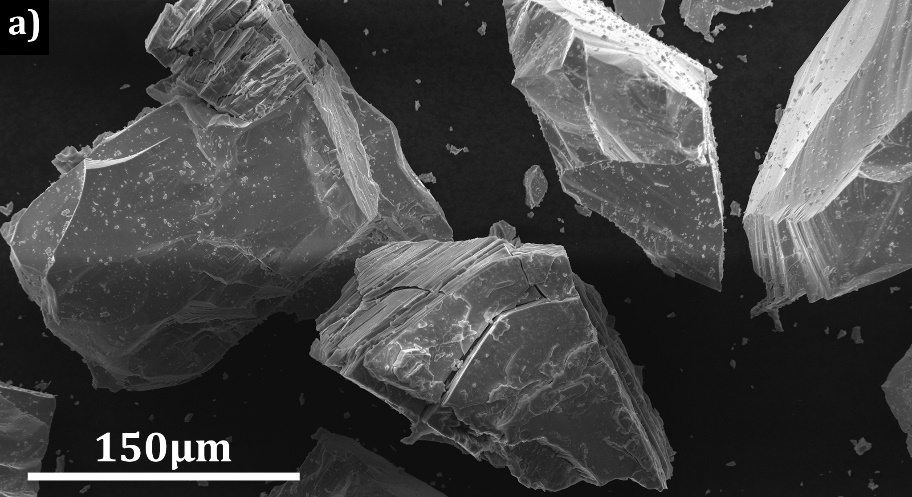


**Figure S4.** FT-IR spectra of SiH with NaOH treatment of CaSi_2_ (purple line) and without (Black line).

**Figure S5.** X-Ray photoelectron spectroscopy (XPS) high-resolution scans of GeH a) Cl 2p and SiH b) Cl 2p and c) F 1s.


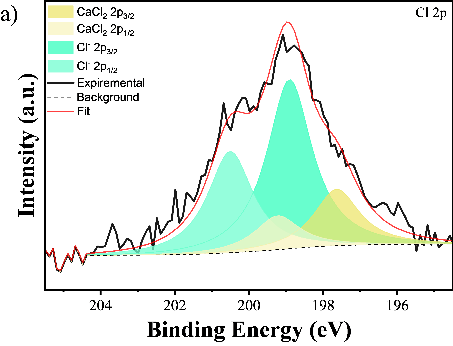

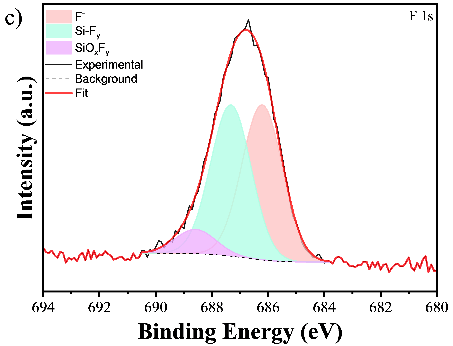

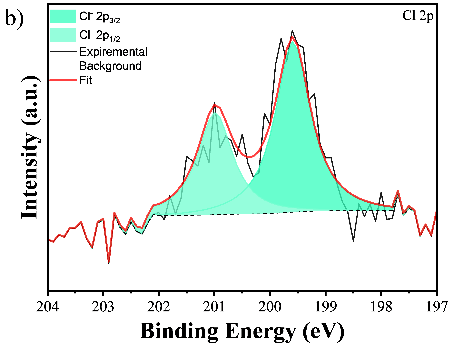

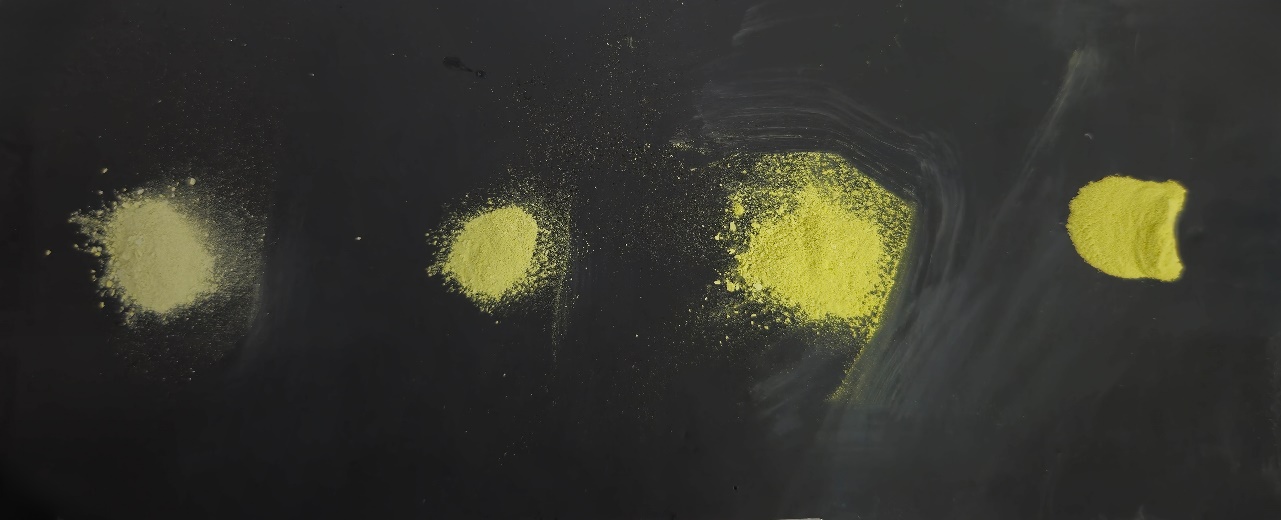


Light exposed

under vacuum

No Light exposed

open atmosphere

No Light exposed

under vacuum

Fresh prepared sample

**Figure S6.** SiH samples stored (for 45 days) in various conditions.

**Figure S7.** XRD of GeH comparing a fresh synthesised batch and the material after 10 days and 45 days storage in ambient conditions.

**Note S1.** Synthesis optimization procedure

XRD was employed to optimize the reaction time. A shift in peak positions was observed corresponding to the successful removal of Ca atoms from CaSi_2_ and formation of SiH in just 15 minutes, with reaction times up to 14 hours yielding comparable results to those obtained within 15 minutes (**Figure S6a**). Additionally, we explored synthesis using different fluoride salts for the "in-situ" HF generation. Both lithium fluoride (LiF) and sodium fluoride (NaF) at equivalent molar ratios were found to result in SiH formation but XRD analysis revealed a shift in the (001) peak for samples prepared with LiF compared to NaF (**Figure S6b**), suggesting enhanced intercalation facilitated by smaller Li^+^ ions. The molarity of HCl was identified as a critical parameter for determining the d-spacing of the (001) plane; lower HCl molarities (6 M) resulted in smaller d-spacing, indicative of milder etching of Ca (**Figure S6c**). Treatment of the precursor with NaOH proved essential for removing Si impurities and preventing dilution of acids used for etching, particularly HF. This treatment effectively eliminated bulk-Si in the sample as evidenced by disappearance of its corresponding diffraction peaks in the XRD pattern in **Figure S6d**.

**Figure S8.** XRD Patterns illustrating the optimization of SiH synthesis for a) the effect of reaction time (blue line: 15’, green line: 45’, black line: 1h30’ and red line: 14h), b) the use of different fluoride salts (red line: NaF & black line: LiF), c) HCl molarity (black line: 6 M, red line: 9 M & blue line: 12 M) and d) the importance of NaOH treatment of CaSi_2_ (red line: untreated & black lined: NaOH treated).

| **Polarity** | **Solvent** | **Molecular Formula** |
| --- | --- | --- |
| **Polar Protic** | Ethanol | 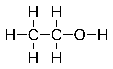 |
|  | Methanol | 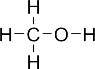 |
| **Polar Aprotic** | N-N-Dimethylformamide | 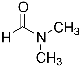 |
|  | Dichloromethane | 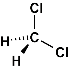 |
|  | Acetonitrile | 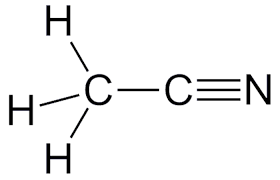 |
|  | N-Methylformamide | 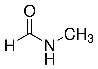 |
|  | 1-Methyl-2-pyrrolidinone | 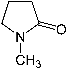 |
|  | Dimethyl Sulfoxide | 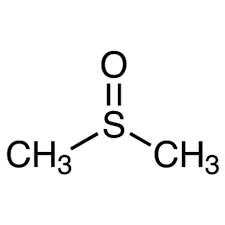 |
|  | Triethylamine | 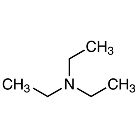 |
| **Non-Polar** | LiCl (0.3M in DI) | 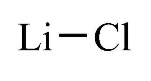 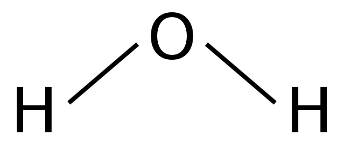 |
|  | n-Hexane | 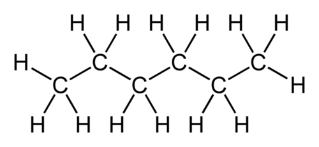 |

| **Polarity** | **Solvent** | **Molecular Formula** |
| --- | --- | --- |
| **Polar Protic** | Deionized Water | 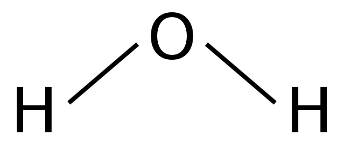 |
|  | Methanol | 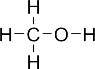 |
|  | Isopropanol | 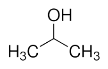 |
|  | Ammonia (in Methanol) | 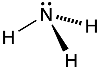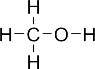 |
| **Polar Aprotic** | Acetonitrile | 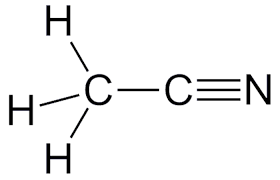 |

**Table S2.** The solvents that were used for SiH solubility test.

**Table S1.** The solvents that were used for GeH solubility test.

DI water

Methanol

Isopropanol

Acetonitrile

Ammonia in

Methanol


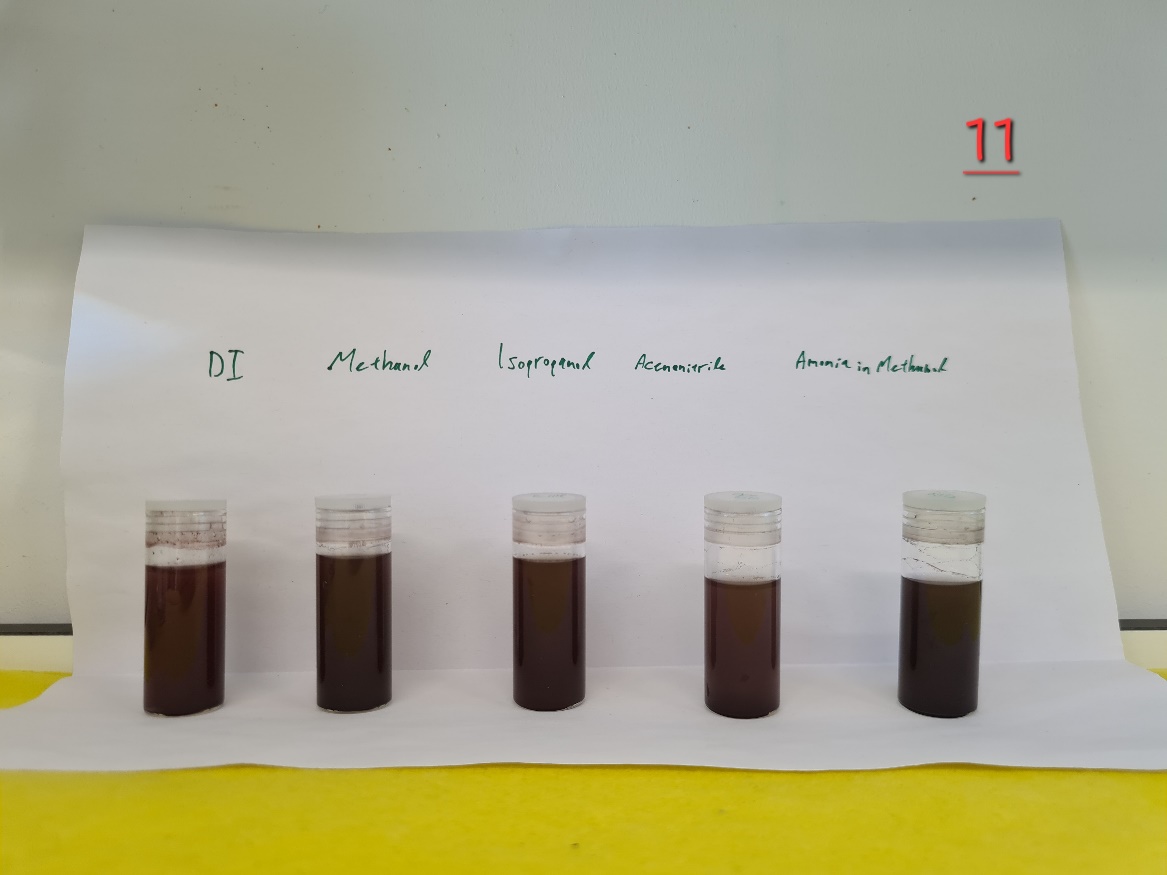


0h


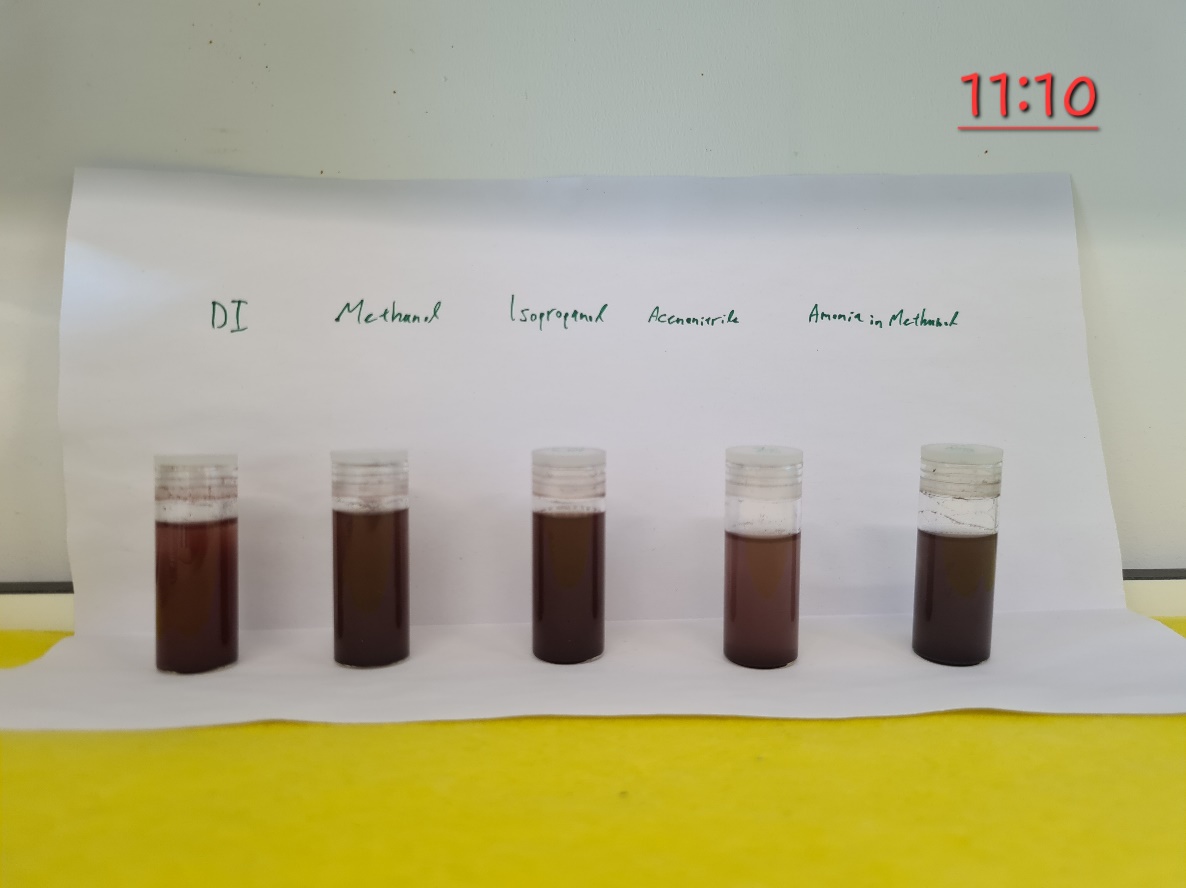


10’


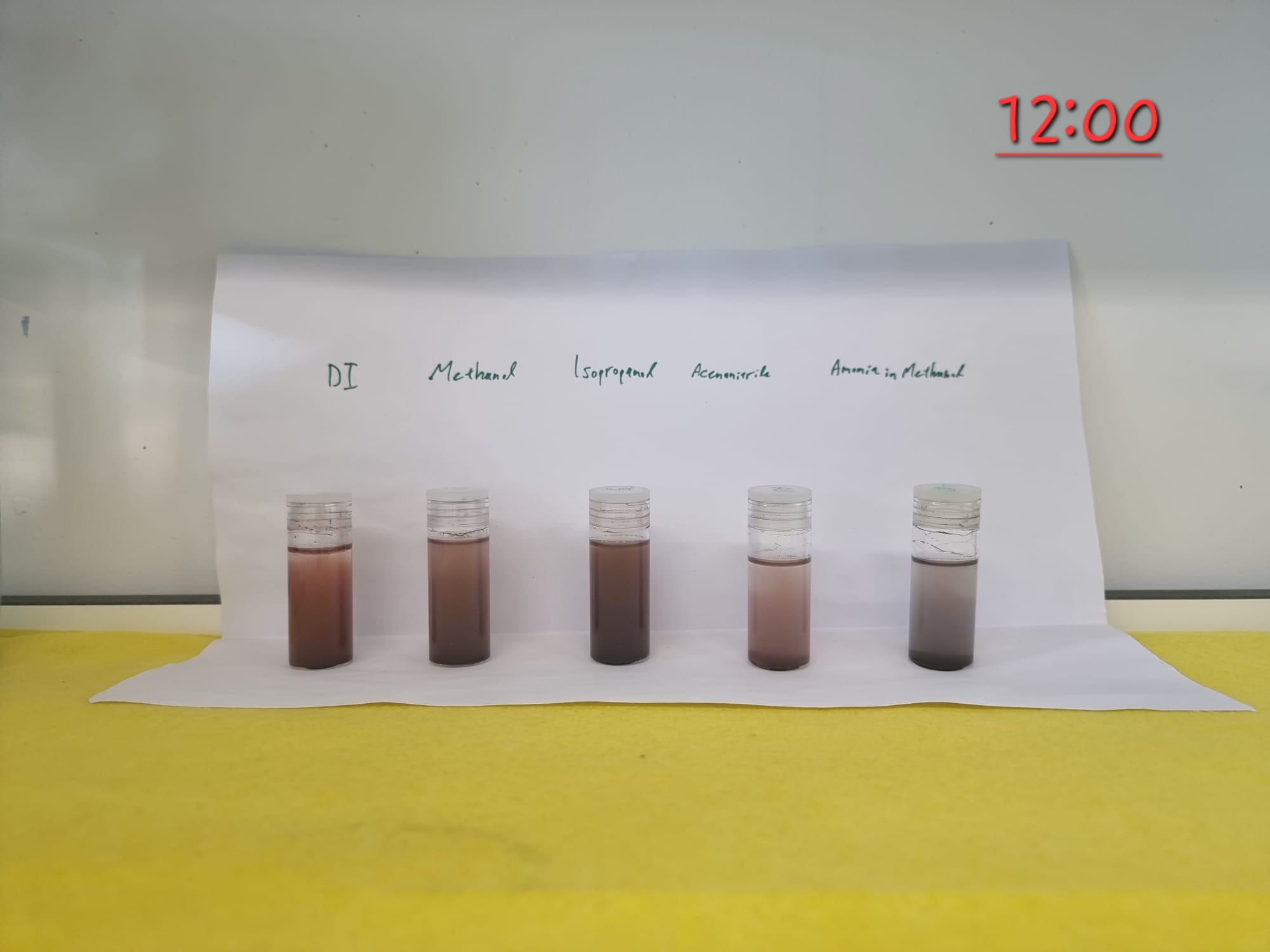


1h


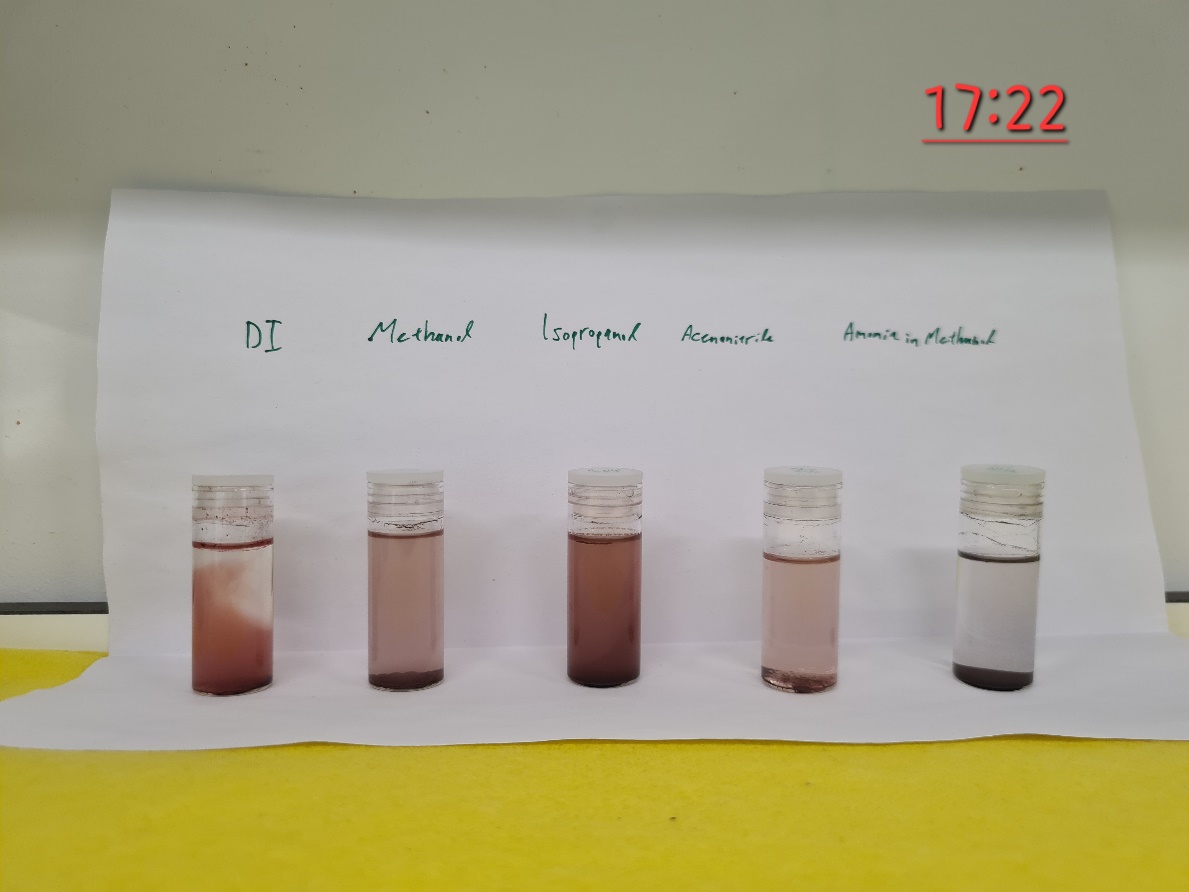


6h

24h


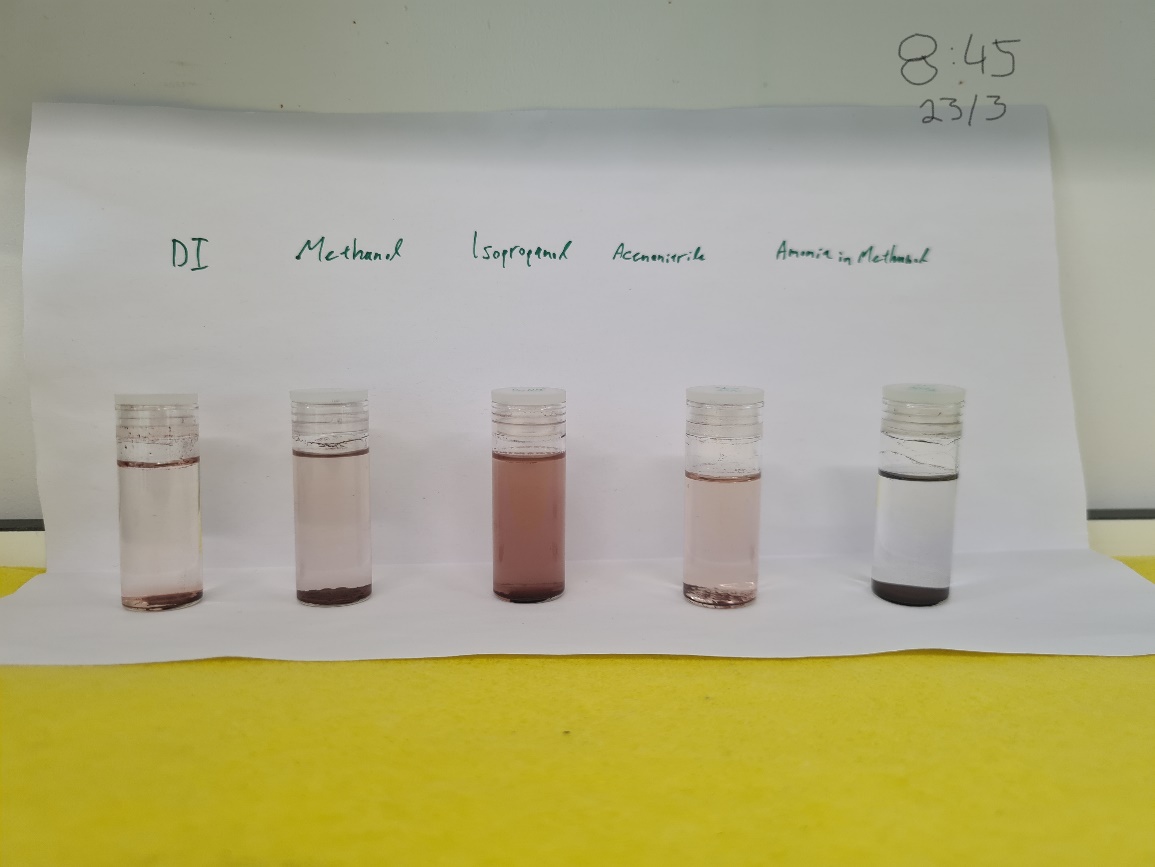


48h


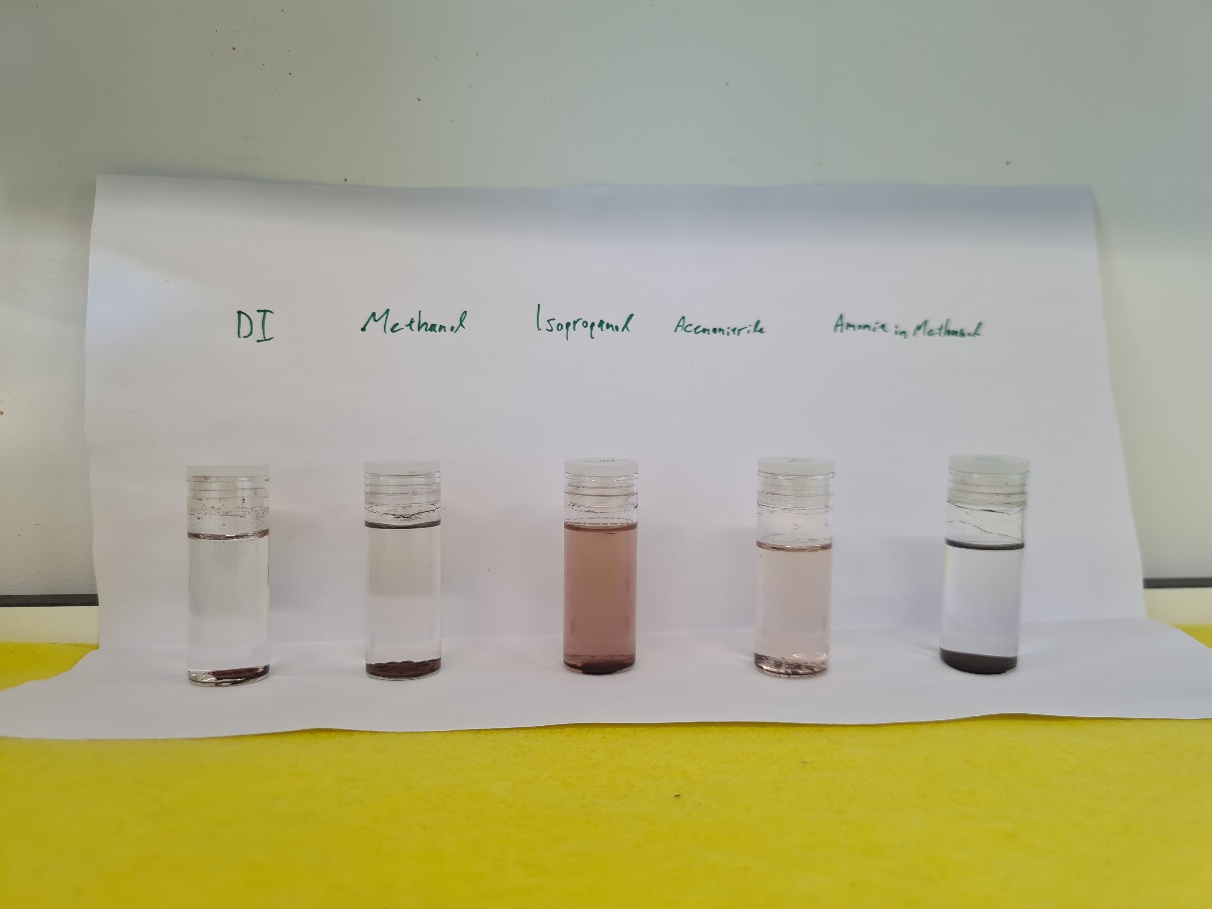


72h


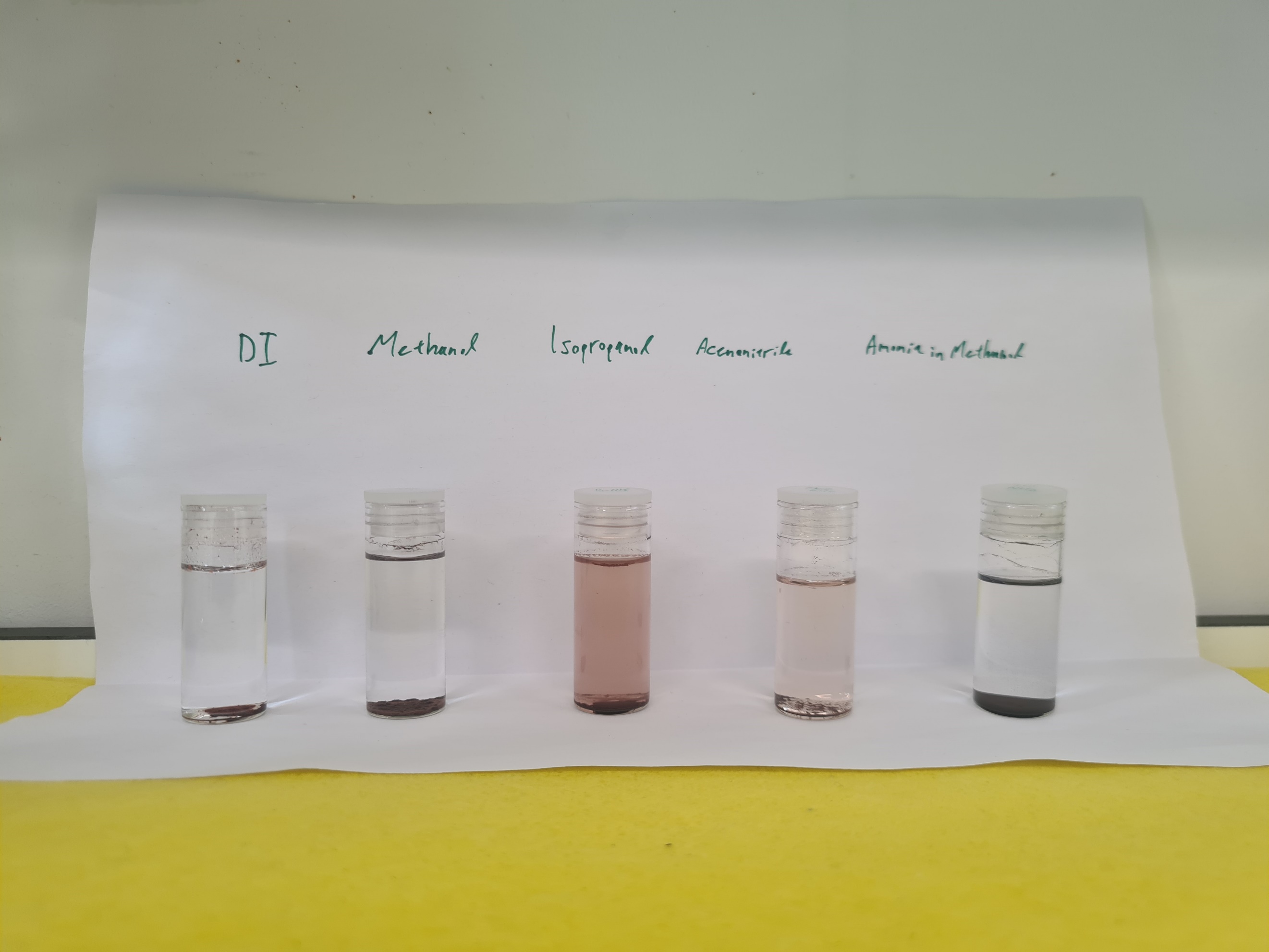


**Figure S9.** Solubility test of GeH in a variety of solvent for 3 days.

N-N-Dimethylformamide

Methanol

Hexane

Triethylamine

Ethanol

Dichloromethane

Acetonitrile

N-Methylformamide

Dimethyl Sulfoxide

1-Methyl-2-pyrrolidinone

LiCl 1g in DI


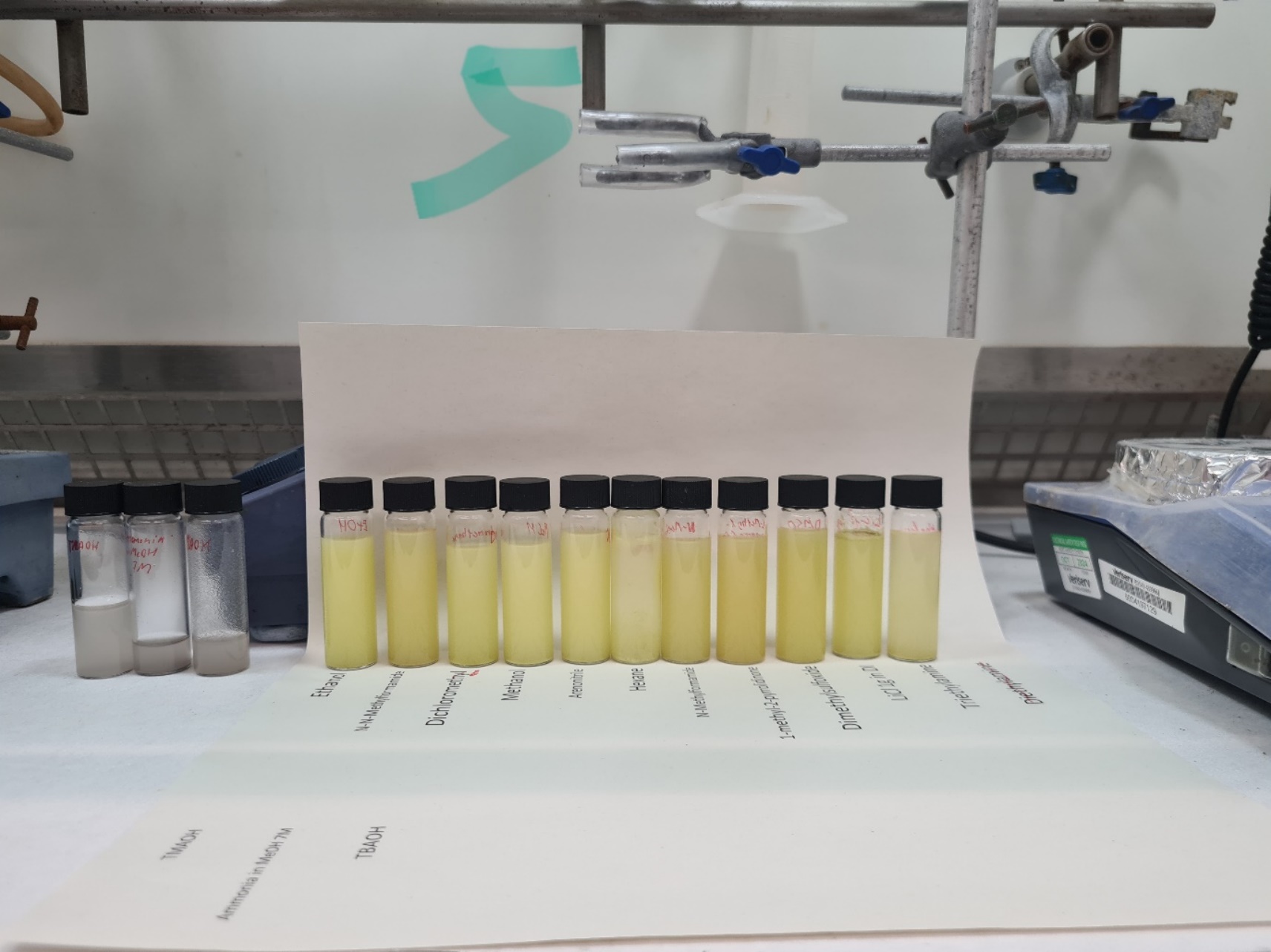


0h


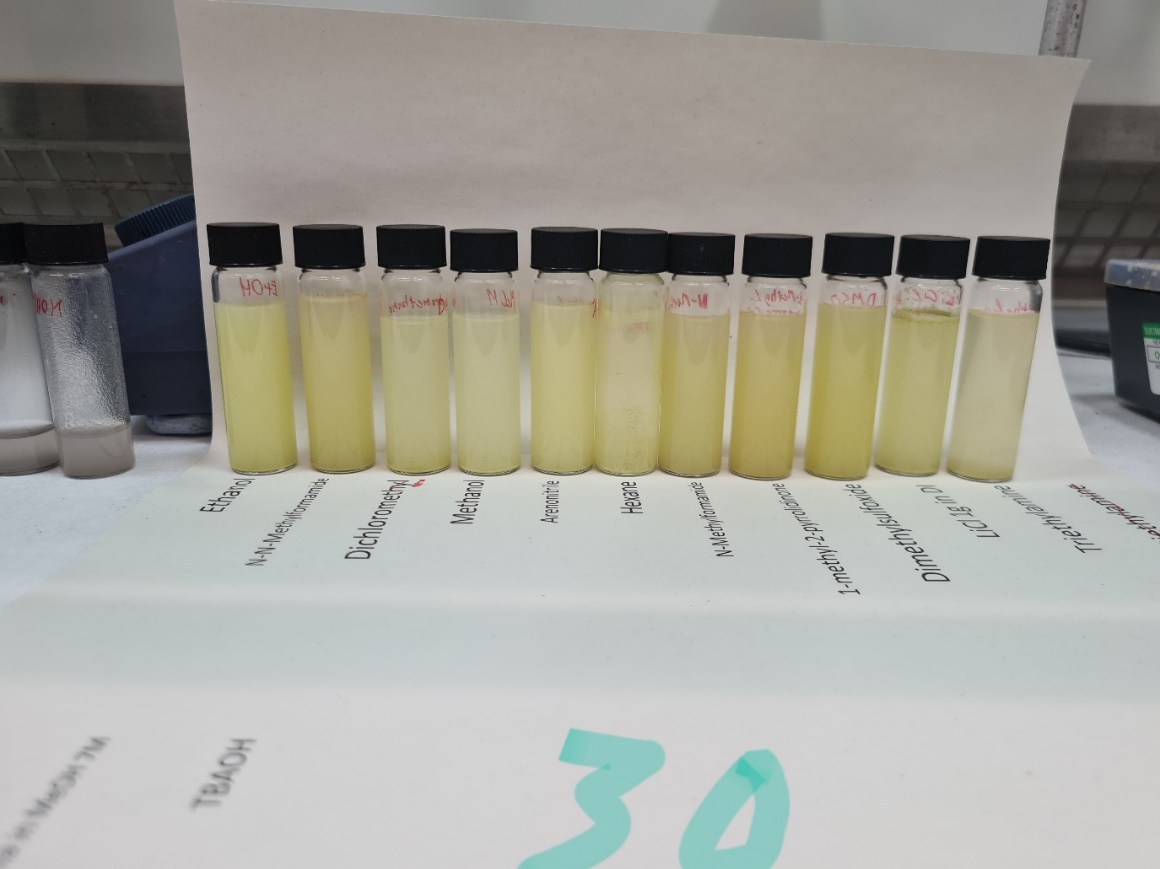


30’


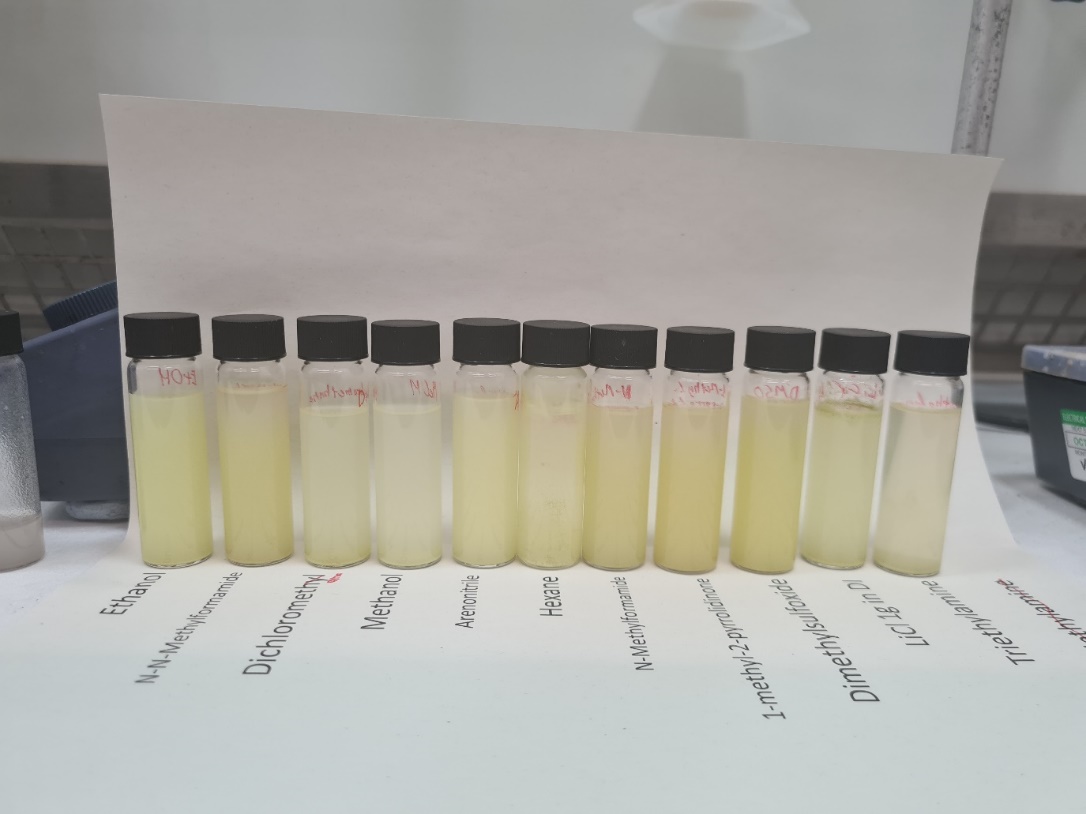


1h


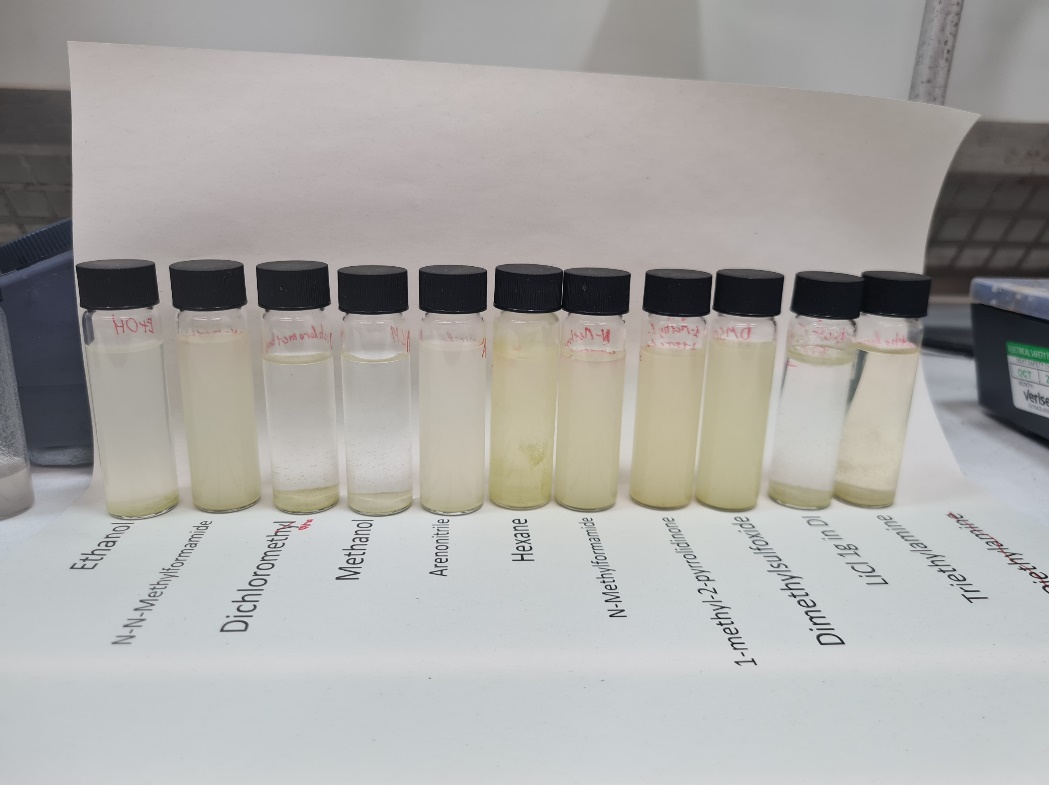


24h


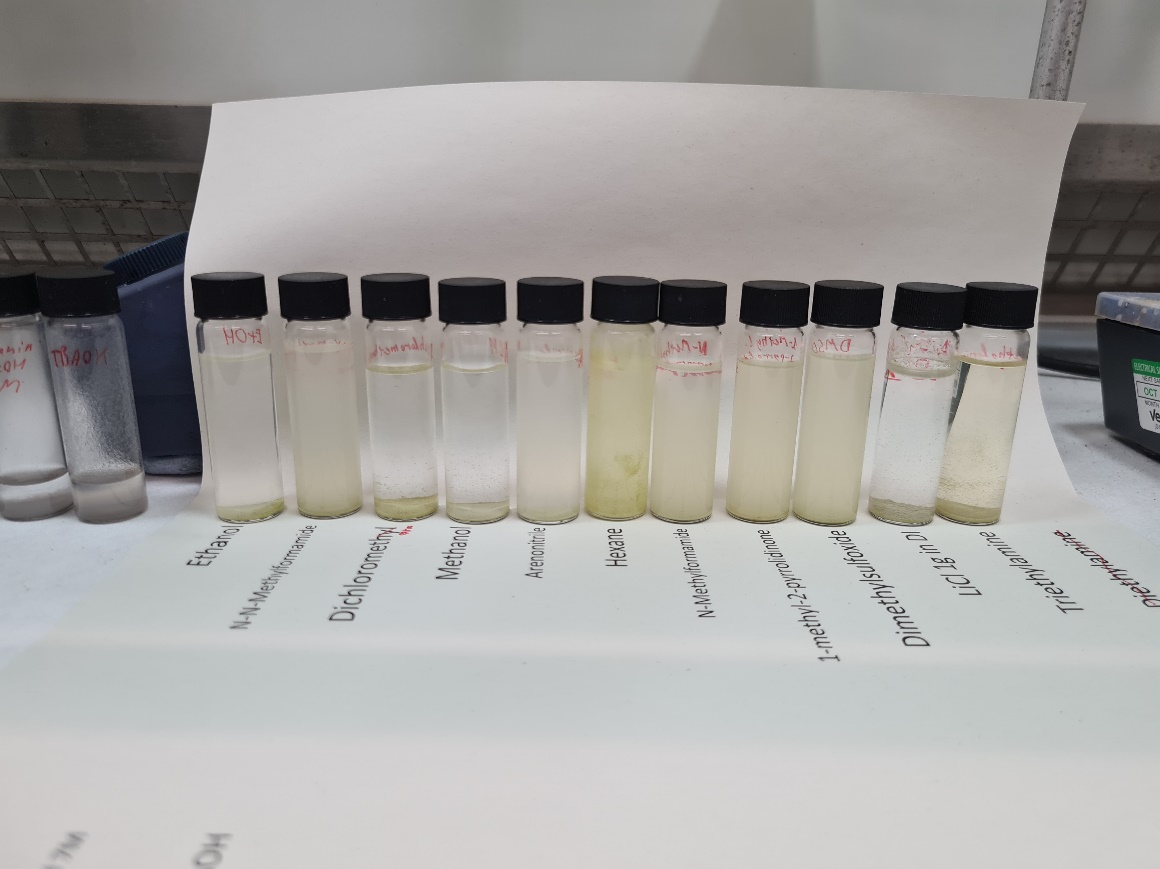


72h

**Figure S10.** Solubility test of SiH in a variety of solvents for 3 days under minimal light exposure.

**Note S2.** Reagents supplementary (Figure S6-S8)

Sodium fluoride (≥97 %) purchased from Thermo Scientific. Dichloromethane (≥99 %) purchased from Fisher Scientific. Hexane (≥95 %) purchased from Sigma-Aldrich. N,N-Dimethylformamide (≥99.8 %) purchased from Sigma-Aldrich. 1-Methyl-2-pyrrolidinone (≥99 %) purchased from Honeywell. Triethylamine (≥99 %) purchased from Sigma-Aldrich. N-Methylformamide (≥99 %) purchased from Sigma-Aldrich. Tetrabutylammonium hydroxide in Methanol (1 M) purchased from Sigma-Aldrich. Tetramethylammonium hydroxide in H_2_O (25 %) purchased from Sigma-Aldrich. Dimethyl sulfoxide (≥99.9 %) purchased from Supelco. Acetone (ACS) purchased from Sigma-Aldrich. Sodium fluoride (≥97 %) purchased from Thermo Scientific.

**Figure S11.** TEM images and the corresponding SAED patterns of amorphous areas of flakes of (a, b) GeH and (c,d) SiH. The circles in a and c indicate the location of the SAED analysis for both flakes.


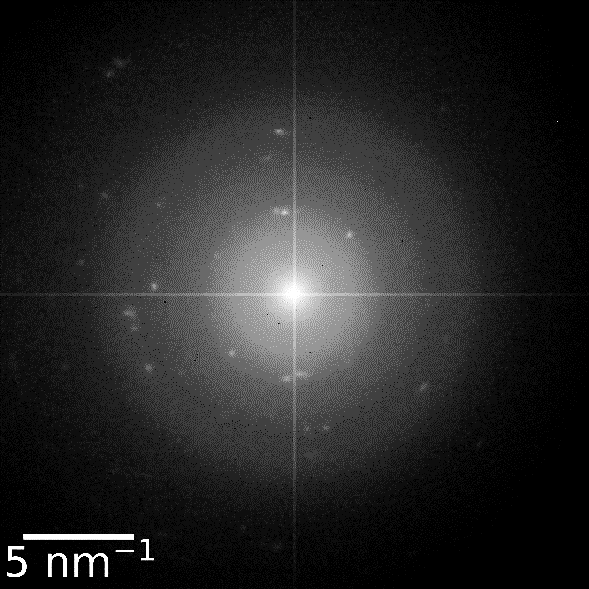

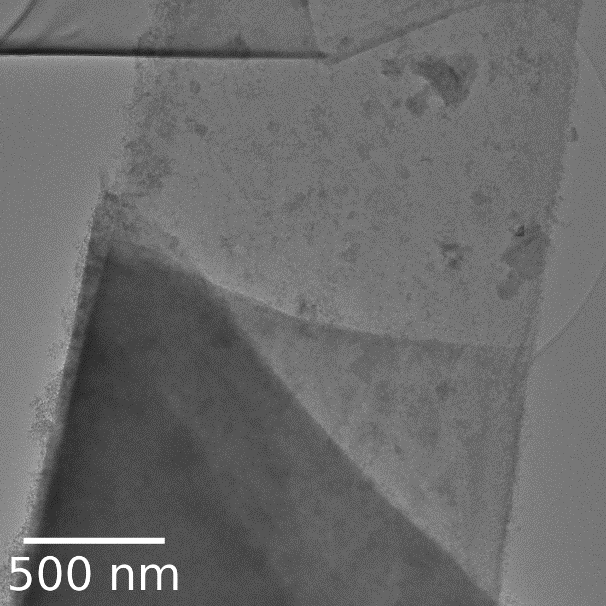

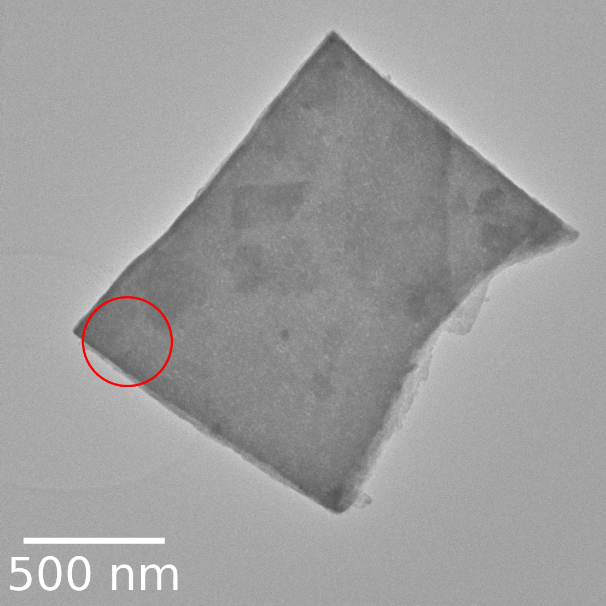

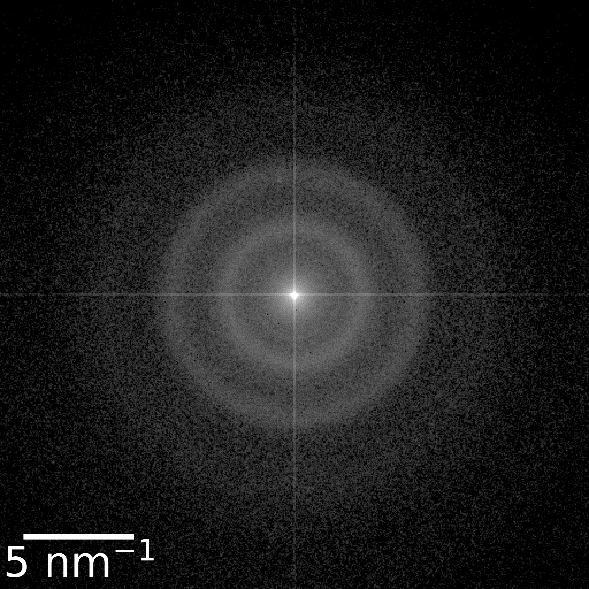


a)

d)

c)

b)


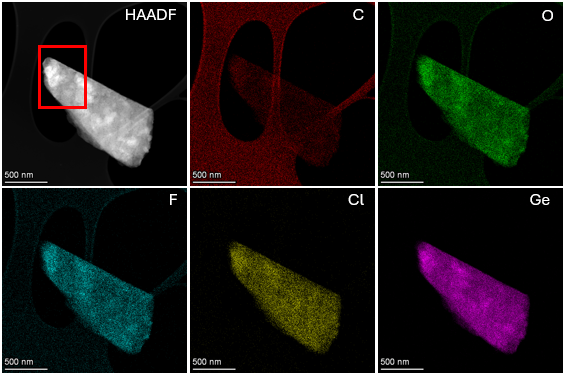


**Figure S12.** STEM EDS elemental maps of GeH flake. Red inset shows region from which the EDS sum spectrum shown in Figure 3c was acquired.


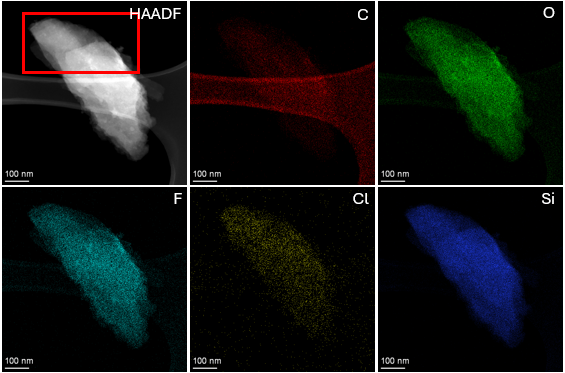


**Figure S13.** STEM EDS elemental maps of a SiH flake. Red inset shows region from which the EDS sum spectrum shown in Figure 3f was acquired.

**Figure S14.** TEM images of GeH flake region from which the crystalline SAED pattern shown in Figure 3(b) was acquired, with the inset FFT of the final image to display the lack of periodic details that could be used to determine details about the crystal structure.


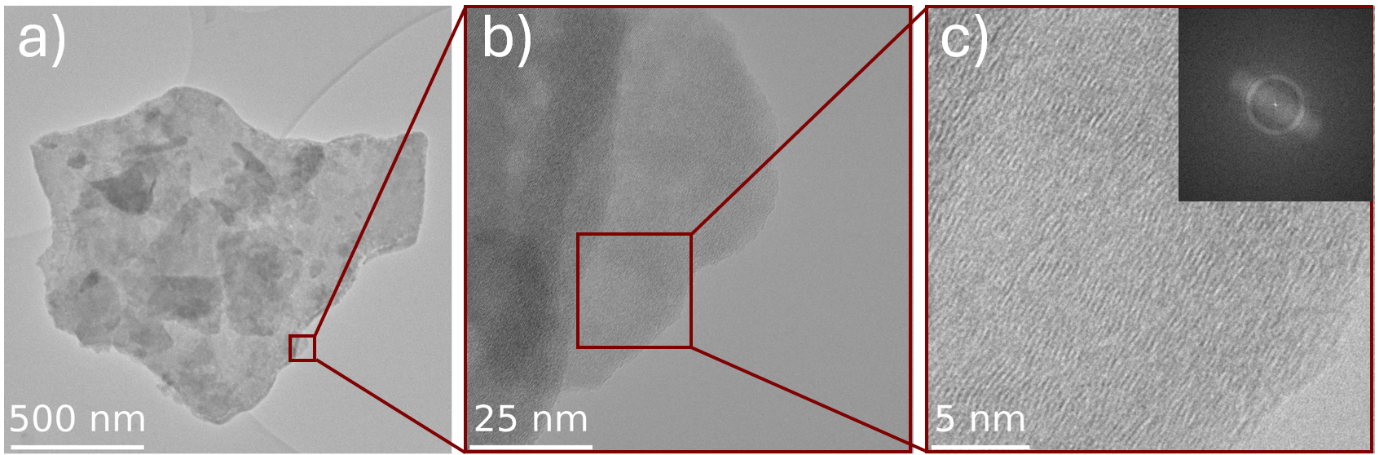

Supplement: Supplementary file 1 — Supporting Information [file SMTD-9-2400964-s001.docx]
